# EhC2B, a C2 domain-containing protein, promotes erythrophagocytosis in *Entamoeba histolytica* via actin nucleation

**DOI:** 10.1371/journal.ppat.1008489

**Published:** 2020-05-04

**Authors:** Aashutosh Tripathi, Megha Jain, Mintu Chandra, Sameena Parveen, Rupali Yadav, Brett M. Collins, Sankar Maiti, Sunando Datta

**Affiliations:** 1 Department of Biological Sciences, Indian Institute of Science Education and Research, Bhopal, India; 2 Institute for Molecular Biosciences, University of Queensland, St Lucia, Australia; 3 Department of Biological Sciences, Indian Institute of Science Education and Research, Kolkata, India; University of Geneva, SWITZERLAND

## Abstract

Remodelling of the actin cytoskeleton in response to external stimuli is obligatory for many cellular processes in the amoebic cell. A rapid and local rearrangement of the actin cytoskeleton is required for the development of the cellular protrusions during phagocytosis, trogocytosis, migration, and invasion. Here, we demonstrated that EhC2B, a C2 domain-containing protein, is an actin modulator. EhC2B was first identified as an effector of EhRab21 from *E*. *histolytica*. *In vitro* interaction studies including GST pull-down, fluorescence-based assay and ITC also corroborated with our observation. In the amoebic trophozoites, EhC2B accumulates at the pseudopods and the tips of phagocytic cups. FRAP based studies confirmed the recruitment and dynamics of EhC2B at the phagocytic cup. Moreover, we have shown the role of EhC2B in erythrophagocytosis. It is well known that calcium-dependent signal transduction is essential for the cytoskeletal dynamics during phagocytosis in the amoebic parasite. Using liposome pelleting assay, we demonstrated that EhC2B preferentially binds to the phosphatidylserine in the presence of calcium. The EhC2B mutants defective in calcium or lipid-binding failed to localise beneath the plasma membrane. The cells overexpressing these mutants have also shown a significant reduction in erythrophagocytosis. The role of EhC2B in erythrophagocytosis and pseudopod formation was also validated by siRNA-based gene knockdown approach. Finally, with the help of *in vitro* nucleation assay using fluorescence spectroscopy and total internal reflection fluorescence microscopy, we have established that EhC2B is an actin nucleator. Collectively, based on the results from the study, we propose that EhC2B acts like a molecular bridge which promotes membrane deformation via its actin nucleation activity during the progression of the phagocytic cup in a calcium-dependent manner.

## Author summary

*Entamoeba histolytica* is an enteric protozoan parasite. It causes a wide range of infections such as diarrhoea, liver abscess, and in severe condition, invades the brain. For the successful completion of the amoebic pathogenesis, there is a remarkable change in amoebic cytoskeleton as soon as the parasite adheres to the host extracellular matrix. However, till date, only very few amoebic proteins have been reported to regulate the cytoskeleton dynamics and EhRab21 is one among them. In this study, for the first time, we have identified an effector of EhRab21, namely EhC2B, and have established its role in phagocytosis and actin nucleation. EhC2B is a member of C2 superfamily and binds to the lipids in the presence of calcium. In the amoebic trophozoites, EhC2B localises to pseudopodal membrane protrusions as well as advancing tips of the phagocytic cup. It also catalyses the actin polymerization via nucleation of actin monomer, thereby, promoting membrane deformation. Thus, EhC2B acts like a link between calcium signalling and cytoskeleton remodelling with implication on phagocytosis.

## Introduction

*Entamoeba histolytica*, a highly motile and pathogenic parasite, is an etiological agent of amoebic dysentery and liver abscesses in humans. It invades the mucosal barriers of the intestinal epithelium and, in turn, spreads into the extraintestinal organs, resulting in amoebiasis. This infectious disease is characterized by extensive destruction of human tissues caused by killing and phagocytosis of human cells [1]. The importance of phagocytosis in amoebiasis is prominent since erythrophagocytosis serves as the marker for the clinical diagnosis of *E*. *histolytica* [2]. Moreover, amoebic mutants deficient in phagocytosis exhibited reduced virulence *in vivo* [3].

The high rate of phagocytosis in *E*. *histolytica* marks it as a unique organism to study the signalling cascade that is activated after attachment of the phagocytic cargo to the cell surface. One of the major objectives of this signalling pathway is to generate the force for the uptake of the cargo, and this is done through reorganisation of the cytoskeleton [4]. Although this pathway has been characterized in many other systems, the molecular mechanism of the same is still largely unknown in *E*. *histolytica*. In some of these model systems, the pathways coupling initiation of phagocytosis to actin dynamics have also been laid out quite extensively [5–7].

It is generally accepted that, during phagocytosis, the pseudopods extend along the target particle and pull it into the cell body. Extension of pseudopods is also a basic mechanism of cell motility for some eukaryotic cells, including *E*. *histolytica*. Multiple lines of evidence suggest that pseudopod extension is driven by actin polymerization beneath the plasma membrane [8] and the actin filaments are concentrated in these pseudopods and the cytoplasm surrounding phagosomes [9]. Subsequently, this would require the participation of a large number of actin-associated proteins. These include actin nucleating, filament severing, end-capping bundling, membrane tethering, and scaffolding proteins [10–15]. Biochemically, pseudopods which are commonly used for the membrane protrusions while the cell is migrating on a substratum is reported to be distinct from the membrane extensions of the phagocytic cup, although morphologically they are similar [16].

Recently, an alternative process of cell killing and internalization has been identified in *E*. *histolytica*, which is termed as trogocytosis [17]. It should be pointed out that only alive target cells undergo trogocytosis, whereas dead target cells are taken up by phagocytosis by the parasite [17]. The process of trogocytosis involves ingestion of several fragments of the live target cells in multiple steps. It also results in an elevation of Ca^2+^ in the cytosol, loss of plasma membrane integrity and eventually death of these cells [17]. Similar to the nature of the phagocytic process, even trogocytosis requires the extending pseudopods to serve as the arms of trogocytic cups, and thus, in turn, entails recruitment of dynamic cytoskeleton.

Ca^2+^ mediated signalling is a critical factor for phagocytosis, trogocytosis as well as regulation of cytoskeleton in several amoebic cellular processes including cell motility, adhesion to fibronectin and cytolytic activity [18–21]. *E*. *histolytica* genome encodes a large repertoire of (approximately 27) calcium-binding proteins (EhCaBPs) [22]. A handful of them, such as EhC2PK, EhCaBP1, EhCaBP3, and EhCaBP5, have been shown to act during the initial stages of phagocytosis [23–25]. EhCaBP1 is involved in the initiation of erythrophagocytosis along with EhC2PK, a C2 domain-containing protein Kinase [23]. EhC2PK accumulates at the site of RBC attachment in a Ca^2+^ dependent step and recruits EhCaBP1, which in turn brings actin filaments resulting in the initiation of phagocytosis [23]. EhCaBP6 is another calcium-binding protein which modulates microtubule dynamics and regulates cell proliferation [26]. Similarly, CaBPs like EhCaBP3 and EhCaBP5 bind to the motor proteins myosin 1A and myosin 1B, respectively. These CaBPs also regulate the closure of the phagocytic cup during the late stages of phagocytosis [24,25,27]. Like EhC2PK, EhC2A is the only other well established C2 domain-containing protein in *E*. *histolytica*. EhC2A mediates transcriptional regulation of URE3-BP by anchoring it to the phosphatidylserine in the amoebic plasma membrane [28]. It has also been associated with phagosomes [29].

An earlier study from our laboratory has suggested that EhRab21, a member of the Rab family of proteins, plays an important role in host tissue invasion by promoting actin dot formation [30]. These actin dots are structurally and functionally similar to ‘invadosomes’ in the mammalian cell lines and contribute to the virulence of this parasite [31]. “Invadosome” is a common term for podosomes (generally used for normal cells) and invadopodia (generally used for cancer cells). These intricate molecular structures are well known to pair actin polymerization with proteolytic activity to coordinate extracellular matrix remodelling and invasion processes [32]. Further, these invadosomes like actin dots have been characterised as protease secretion apparatus using amoebic cell surface proteases [32].

In the current study, we have identified and established EhC2B, a C2 domain-containing protein, as an effector for EhRab21 under *in vitro* condition. Next, EhC2B demonstrated calcium-dependent preferential binding to phosphatidylserine in addition to a few other phosphoinositides. In the amoebic trophozoites, EhC2B localised at the pseudopods and the tips of the phagocytic cups as well as the trogocytic cups. Downregulation of EhC2B resulted in abrogation of erythrophagocytosis. Finally, our results indicate that EhC2B directly binds to G-actin and stimulates F-actin assembly via actin nucleation, consequently, highlighting the importance of EhC2B in amoebic virulence.

## Results

### Identification of EhC2B, a C2 domain-containing protein, from *E*. *histolytica* as an interacting partner of EhRab21

Although the function of EhRab21 is already known [30], binding partners for the GTPase remained unidentified. We employed GST pull-down assay coupled with mass spectrometry to identify EhRab21 effectors. The GSTEhRab21 fusion protein was immobilised on glutathione-sepharose beads. The nucleotide bound to the EhRab21 was exchanged with an excess of GDP or GppNHp (non-hydrolysable GTP analogue) in the presence of EDTA to prepare GDP-GSTEhRab21 or GppNHp-GSTEhRab21 complex, respectively. The immobilised proteins were then incubated with amoebic cell lysate for 2 hours at 4°C, and the unbound proteins were removed by stringent washes. The mass spectrometric analysis of the column elutes identified EhC2B, as a GppNHp specific interacting partner of EhRab21.

**Fig 1:**
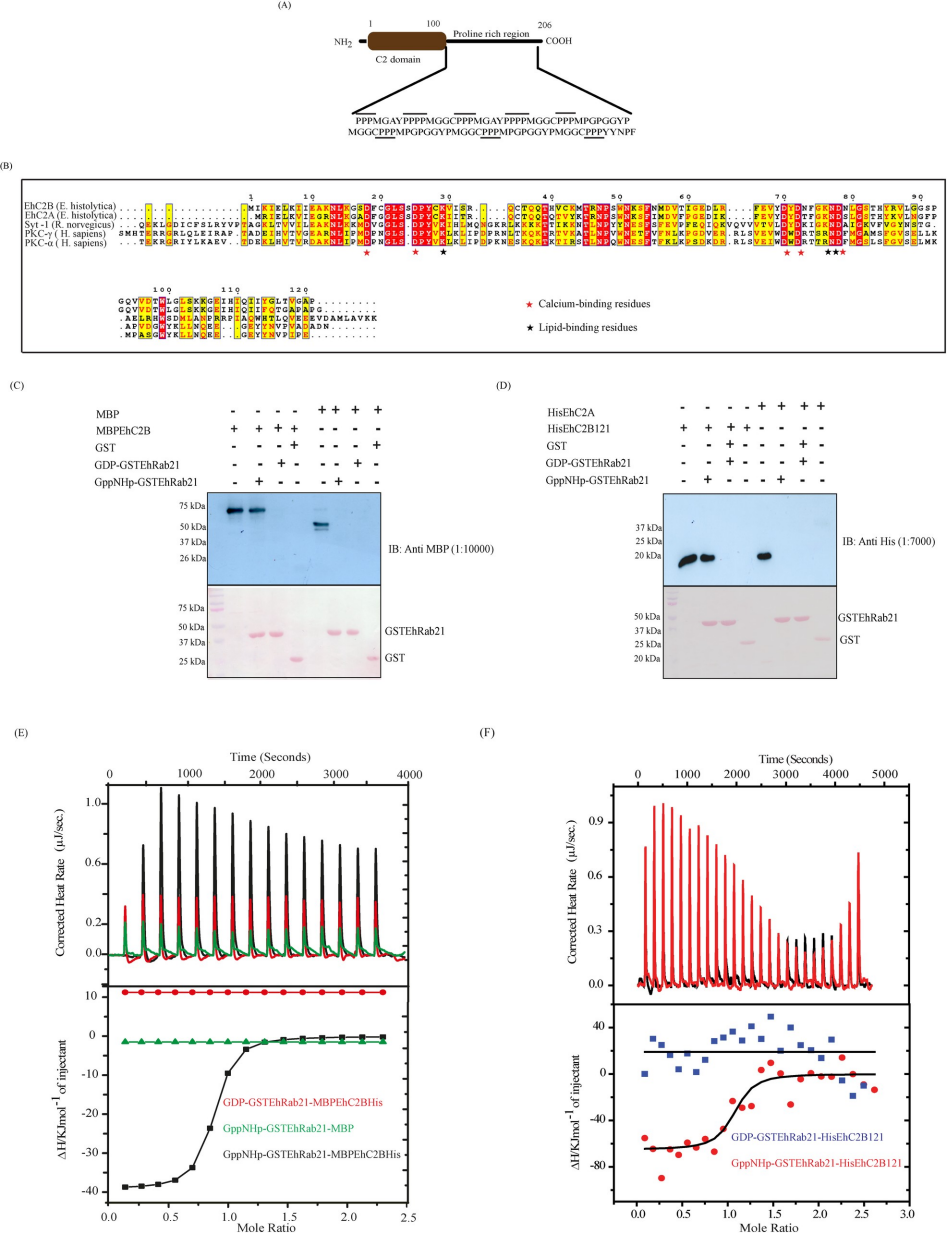
(A) Domain architecture of EhC2B. The Pfam database (https://pfam.xfam.org/) search of EhC2B has predicted an N-terminal C2 domain and a proline-rich region at the C-terminal end of the protein. In the C-terminal region, each proline amino acid is lined. (B) Sequence alignment of the C2 domain of EhC2B against the C2 domain of other proteins (*E*. *histolytica* EhC2A, *R*. *norvegicus* Syt1, *H*. *sapiens* PKC-α and PKC-γ). The conserved calcium and lipid-binding residues in the proteins are indicated by red and black asterisks, respectively. (C) The EhC2B binds to the EhRab21 preferentially in the presence of GppNHp. One micromolar of recombinant purified GSTEhRab21 protein was immobilised on the glutathione-sepharose beads and incubated with 5 μM of EhC2B full-length protein, fused with the MBP at the N-terminal and His-tag at C-terminal end for one hour at 4°C. After the multiple washes, the reaction mixture was resolved by SDS-PAGE, and the binding of EhC2B to GSTEhRab21 was detected by immunoblotting using an anti-MBP antibody, as shown in the upper panel. The lower panel shows the ponceau staining of the bait proteins. (D) The N-terminal C2 domain binds to the EhRab21 independent of C-terminal proline-rich region. The glutathione sepharose beads immobilised GSTEhRab21 protein was incubated with HisEhC2B121 or EhC2A (C2 domain) for 1 hour. The binding between both the proteins was detected by immunoblotting using an anti-His antibody. The bait proteins are shown by ponceau staining in the lower panel. (E) The EhC2B binds to EhRab21 in the presence of GTP. The upper panel shows the raw ITC data obtained from the titration of 76 μM EhRab21 into 10 μM of full-length MBP fused EhC2B in the presence of GDP (black) or GppNHp (red) whereas MBP is shown by green colour. The lower panel represents the best least-square fit of the integrated heat obtained from the raw data after subtracting the heat of dilution. The square (black) and tringle (red) represent the fitted data in the presence of GppNHp and GDP, respectively. The red circle shows the fitted data of MBP. (F) The C2 domain (EhC2B121) binds and preferentially interacts with GppNHp bound EhRab21. The thermogram presents titration of 76 μM EhRab21 with 10 μM of HisEhC2B121 in the presence of GppNHp (black), and GDP (red) is shown in the upper panel. The lower panel represents the best square fit of the resultant integrated heat after subtracting the heat obtained from the dilution.

*In silico* analysis of EhC2B amino acid sequence in the Pfam database predicted the presence of an N-terminal C2 domain (1–100 amino acids) and a proline-rich region at the C-terminal end of the protein (Fig 1A). EhC2B is found to be the closest homologue of EhC2A by BLAST database search of its amino acid sequence (http://amoebadb.org/amoeba/). The amino acid sequence of EhC2B shares 76% identity with EhC2A. Both the proteins contain a C2 domain and are the members of C2 superfamily. In an earlier study, Petri *et al*. had demonstrated that EhC2A is a Ca^2+^ binding protein which helps in the recruitment of URE3-BP, a transcription factor to the plasma membrane in response to the rise in the intracellular Ca^2+^ level [28].

We also aligned the amino acid sequence of the C2 domain of EhC2B against the C2 domains of some other well-characterised C2 superfamily members, belonging to other organisms (Fig 1B). The alignment, as shown in Fig 1B, reveals the presence of the conserved lipid and Ca^2+^ binding residues in EhC2B.

To validate the results from the proteomics study and determine whether EhC2B directly interacts with the GTP bound EhRab21, we performed GST pull-down assay using recombinant EhRab21 and EhC2B. The GSTEhRab21 protein was immobilised on glutathione sepharose beads. The immobilised protein was then incubated with an excess of GDP and GppNHp to have GDP-EhRab21 and GppNHp-EhRab21, respectively. Next, the affinity-purified MBP fused full-length EhC2B protein (S1 Fig) was incubated with immobilised GSTEhRab21 for one hour at 4°C on a rotamer. The unbound proteins were removed by multiple washes. Afterwards, the beads were subjected to SDS-PAGE followed by Western blot using an anti-MBP antibody. To rule out the possibility of any nonspecific interaction, we have used MBP as control and checked its binding with EhRab21 in the presence of GppNHp/GDP. The pull-down data reveals that EhC2B binds to the EhRab21 preferentially in the presence of GppNHp (Fig 1C). Next, to understand whether the N-terminal C2 domain of EhC2B alone can bind to the EhRab21 or not, we have purified its C2 domain (1–121) with an N-terminal hexahistidine tag and used it in the pull-down assay (S2 Fig). The recombinant protein hereafter is referred to as EhC2B121. It includes the N-terminal C2 domain and 20 additional amino acids at the C-terminal. The recombinant HisEhC2B121 was incubated with the nucleotide-bound immobilised GSTEhRab21 proteins. Unbound HisEhC2B121 was removed by the stringent washes, and the binding between both the proteins was detected by immunoblotting using an anti-His antibody as detailed in the Methods section (Fig 1D). Similar to the full-length protein, HisEhC2B121 preferentially interacted with EhRab21-GppNHp (Fig 1D). The interaction between HisEhC2B121 (C2 domain) and EhRab21 is specific as otherwise, the highly identical (76%) C2 domain of EhC2A did not bind to EhRab21 (Fig 1D). This result led us to conclude that EhC2B is a nucleotide-dependent effector of EhRab21 *in vitro*, and its C2 domain alone is sufficient to bind to the GTPase.

Fluorescence-based assay further supported the nucleotide-dependent interaction for effector binding [33]. This assay is based on the fluorescence property of Mant [2'—/3'—O—(N'–Methylanthraniloyl)] conjugated guanine nucleotide, MantGppNHp. EhRab21 was complexed with MantGppNHp by reducing the Mg^2+^ concentration with excess EDTA, such that the GDP that was initially bound to EhRab21 is dissociated and is replaced by the Mant-nucleotide. The excess (unbound) MantGppNHp is then removed by eluting the exchange reaction mixture through a NAP-5 desalting column. The addition of the HisEhC2B121 in an increasing concentration to the MantGppNHp-GSTEhRab21 complex yielded a rise in the Mant emission fluorescence (Excitation 360nm, Emission 440nm) (S3 Fig). To assess the nucleotide dependence of EhC2B, GDP or GppNHp bound EhRab21 was added to the HisEhC2B121 bound MantGppNHp-EhRab21. The enhancement in fluorescence due to the addition of HisEhC2B121 was found to be reversed more effectively by GppNHp-GSTEhRab21 than GDP-EhRab21 (S3 Fig). This observation indicates that EhC2B preferentially binds to GppNHp-EhRab21.

To quantify the strength of the interaction of EhC2B full-length or C2 domain with EhRab21 and to understand its thermodynamic basis, we employed isothermal titration calorimetry (ITC). As shown in Fig 1E and 1F, both MBPEhC2B and HisEhC2B121 bind to the GppNHp-GSTEhRab21 with an association constant (Ka) of 1.24 x10^7^ M^-1^ and 9.6x10^6^ M^-1^, respectively. Overall the EhC2B binding to EhRab21 was spontaneous and is enthalpically driven. Table 1 shows the thermodynamic parameters for the interaction. No detectable interaction was observed between GDP-EhRab21 and MBPEhC2B or HisEhC2B121 re-establishing that EhC2B binds to GppNHp bound EhRab21 and thus, it is a genuine effector *in vitro* (Fig 1E and 1F).

**Table 1:** Thermodynamic parameters for the HisEhC2B121 and EhRab21 interaction in the presence of GTP and GDP.

Protein	Ligand	[EhC2B] (μM)	[Ligands] (μM)	ΔH (KJ mol^-1^)	ΔS (Jmol^-1^ K^-1^)	TΔS (KJ mol^-1^)	ΔG (KJ mol^-1^)	Ka (M^-1^)	n
EhC2B	GppNHp-EhRab21	10	76	-38.6	5.145	1.553	-37.04	1.24x10^7^	1
EhC2B121	GppNHp-EhRab21	10	76	-65.1	-84.7	-25.2	-39.9	9.6x10^6^	1
EhC2B/ EhC2B121	GDP-EhRab21	No Interaction							

### Calcium is essential for the preferential binding of EhC2B with Phosphatidylserine

**Fig 2:**
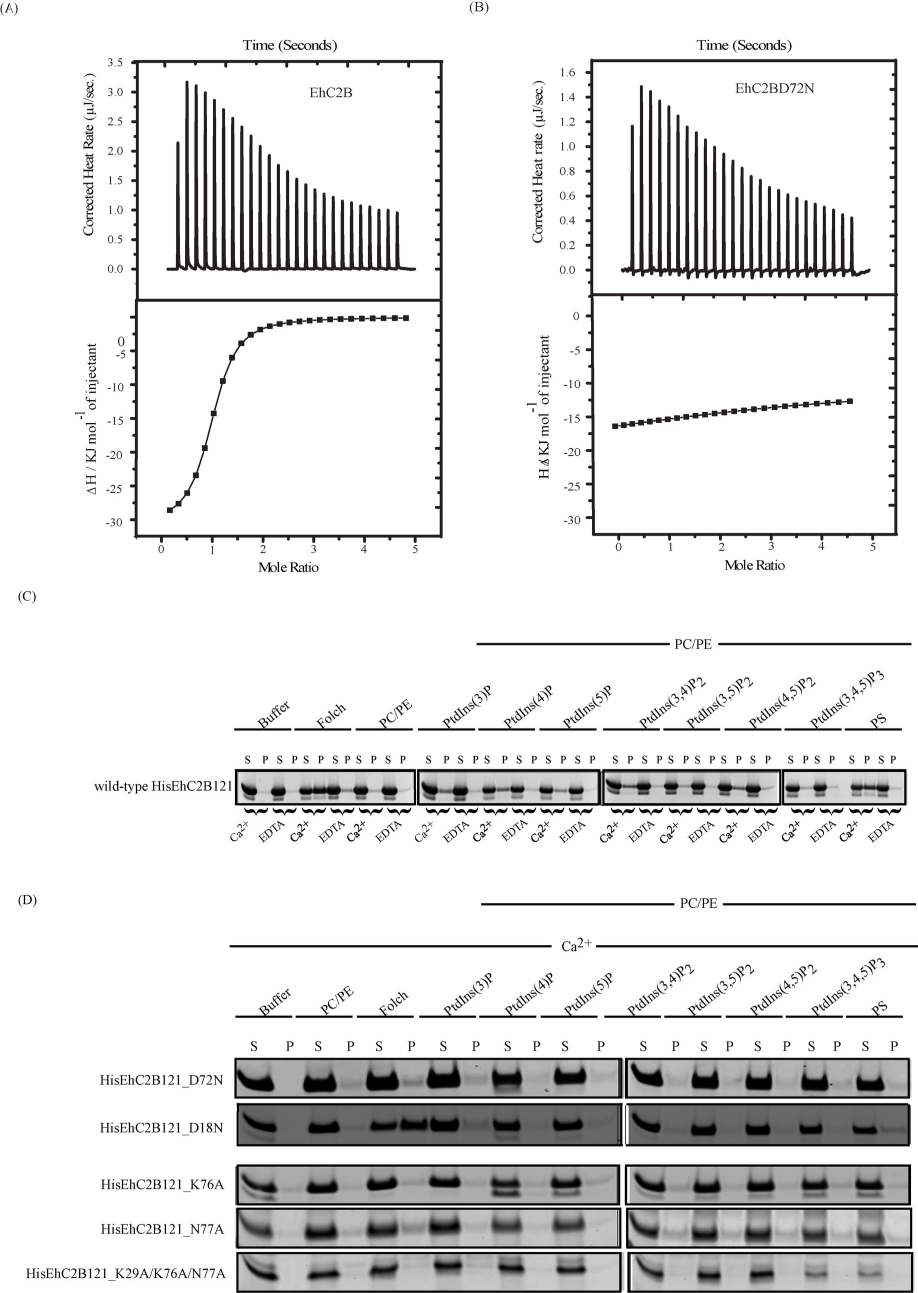
(A) Interaction of EhC2B with calcium. Fifty micromolars of HisEhC2B121 was titrated with 700 μM of Ca^2+^ at room temperature. Top panel shows raw data of interaction and the bottom panel represents the best least-square fit of the integrated normalised data of Ca^2+^ and HisEhC2B121 interaction. (B) Raw data (top) and integrated normalised data (bottom) for 700 μM Ca^2+^ and 50 μM His-EhC2BD72N binding was obtained from the 25 automated injections and fitted into one set of sites model of interaction. (C) Liposome pelleting assay of the wild-type His-EhC2B121, (D) EhC2B calcium-binding mutants (D18N, D72N) and lipid-binding mutants (K76A, N77A, K29A/K76A/N77A). The purified wild-type and mutants HisEhC2B121 were incubated with artificial liposome for 25 minutes at room temperature in the presence of calcium and EDTA. The sample mix was fractionated into pellet and supernatant by high-speed centrifugation analysed to SDS-PAGE.

C2 superfamily proteins are known to interact with lipids in a calcium-dependent manner [34]. To determine whether EhC2B binds to Ca^2+^, we carried out an interaction study using ITC. 50 μM of HisEhC2B121 was titrated with 700 μM of Ca^2+^. The thermodynamic parameters were obtained after fitting the raw data of interaction into the 1:1 binding model (independent model) (Fig 2A). The value of enthalpy (ΔH) and entropy (Δ*S*) are -31KJmol^-1^ and -2Jmol^-1^K^-1^, respectively (Table 2). Our calorimetric study suggested that HisEhC2B121 binds to the Ca^2+^ with an affinity constant of 3.17 x 10^5^ M^-1^ (Table 2). The interaction between HisEhC2B121 and Ca^2+^ is spontaneous, as shown by the negative value of ΔG and is enthalpically favoured (Table 2). We further investigated whether the conserved amino acids, which are known to be crucial for Ca^2+^ binding in other C2 family proteins, do play a similar role in EhC2B (Fig 1B). We mutated D72 residue of EhC2B into N72 and investigated its interaction with Ca^2+^ using ITC [35]. The mutant did not show interaction with Ca^2+^ proving the importance of conserved residue D72 in Ca^2+^ binding (Fig 2B).

**Table 2:** Thermodynamic parameters for the wild-type HisEhC2B121 and calcium interaction.

Protein	Ligand	[EhC2B] (μM)	[Ligands] (μM)	ΔH (KJ mol^-1^)	ΔS (Jmol^-1^ K^-1^)	TΔS (KJ mol^-1^)	ΔG (KJ mol^-1^)	Ka (M^-1^)	n
HisEhC2B121	Ca^2+^	50	700	-31	-2	-0.596	-30	3.17x10^5^	1
HisEhC2B121-D72N	Ca^2+^	No Interaction							

To validate our assay, we have also purified the C2 domain of EhC2A and used it for the ITC to check its interaction with Ca^2+^. Although the calcium-dependent lipid-binding nature of EhC2A has been reported, its direct interaction with the calcium is not shown yet. In our study, we have shown that the C2 domain of EhC2A interacts with calcium directly similar to the HisEhC2B121. The stoichiometry of the interaction is 1:1, and the affinity constant is 4.06 x 10^6^ M^-1^ (S4 Fig).

To determine the EhC2B-lipid interaction, we performed lipid overlay assay and further extended the study by carrying out liposome pelleting assay. In lipid overlay assay, various lipids were spotted onto a nitrocellulose membrane. The membrane was later incubated with HisEhC2B121 in the presence of Ca^2+^. After adequate washes, the membrane was probed for bound HisEhC2B121 by immunoblotting using an anti-His antibody. HisEhC2B121 was found to bind Phosphatidylserine (PS), Phosphatidylinositol (PtdIns) and its variants such as PtdIns (3)P, PtdIns(4)P, PtdIns(5)P, PtdIns(3,5)P2, PtdIns(4,5)P2 (S5A Fig). To find out whether the conserved lipid-binding residues are essential, we mutated these residues and generated a triple mutant (K29A/K76A/N77A) (Fig 1B). The triple lipid mutant did not bind to any of the lipids, as shown by overlay assay (S5B Fig). This confirmed that like other C2 superfamily proteins, the C2 domain of EhC2B also carries the conserved lipid-binding residues. Next, we carried out the liposome pelleting assay to determine lipid preference of EhC2B and its calcium dependence. The wild-type EhC2B protein was incubated with the liposomes, each made using specific lipid molecule, in the presence of Ca^2+^ or EDTA. We also used Folch fraction brain lipids as a control since they indicate broader lipid-binding activities. Folch lipids contain a significant amount of PS and PI(4,5)P2. HisEhC2B121 was incubated either with buffer or PC/PE or Folch liposome. A considerable amount of HisEhC2B121 protein binds to Folch liposome in the presence of Ca^2+^ (Fig 2C). In C2 domain proteins, Ca^2+^ is believed to act as a bridge between negatively charged lipid and protein [36]. Presence of EDTA chelates the Ca^2+^ and thus perturbs the lipid-binding. As a result, we observe more protein in the supernatant fraction (Fig 2C). This result suggests that Ca^2+^ is essential for EhC2B lipid-binding. Similar experiments were performed with other liposomes containing different phosphate variants of PtdIns and PS, respectively. Among various lipids tested, EhC2B binds to PtdIns(3)P, PtdIns(4)P, PtdIns(5)P, PtdIns(3,5)P2, PtdIns(4,5)P2 but preferentially binds to the phosphatidylserine in a Ca^2+^ dependent manner (Fig 2C).

Liposome pelleting assay was also performed using defective calcium mutants of EhC2B (D18N and D72N). Although the Folch/PS binding to D18N mutant remained unaffected, the D72N mutant did not show any significant/detectable binding to either Folch or phosphatidylserine (Fig 2D). This observation suggests that the aspartate (D72) is critical for phosphoinositides mediated interaction in the presence of calcium. As revealed from the amino acid sequence alignment (Fig 1B) and lipid overlay assay (S5A Fig, S5B Fig), the conserved amino acid residues, K76, N77, and K29 would be necessary for lipid-binding. Next, we investigated the lipid-binding property of EhC2B mutants K76A, N77A and K29A/K76A/N77A. The phosphatidylserine binding to all the mutants was diminished when compared to the wild type EhC2B, suggesting that K76 and N77 are essential for the lipid-binding (Fig 2D).

### EhC2B is enriched at phagocytic cups and the membrane protrusions

**Fig 3:**
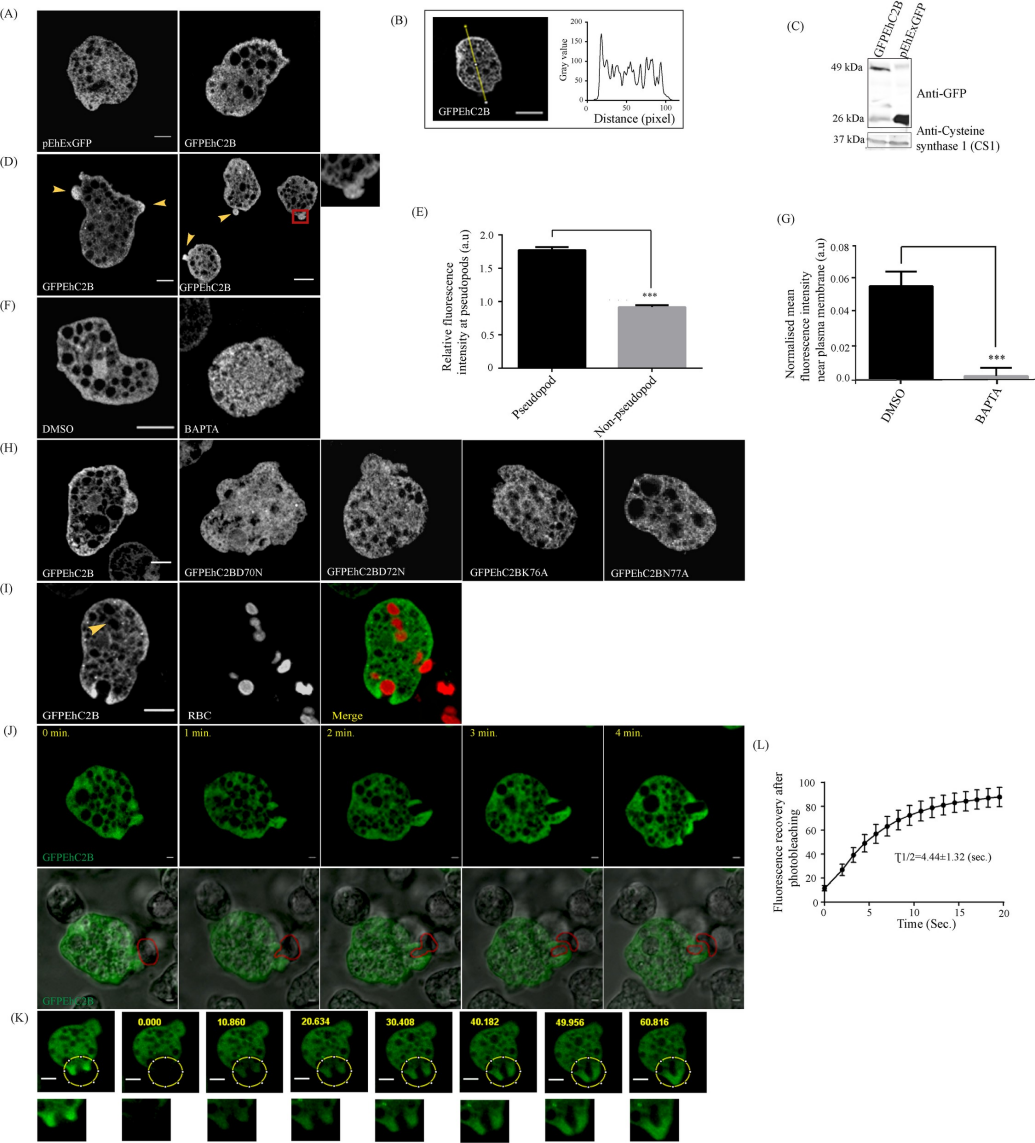
Subcellular localization of EhC2B. (A) pEhEx-GFP (vector control) and pEhEx-GFPEhC2B transfected amoebic trophozoites were grown at 20μg ml^-1^ G418, incubated in glass wells for 15mins at 37°C, fixed and visualised using confocal microscopy. (B) The fluorescence intensity of GFPEhC2B was analysed across the amoebic trophozoites. The line intensity plot shows the change in the fluorescence intensity with the distance. (C) Total cell lysates (400μg) from GFPEhC2B and GFP (vector control) transfected amoebic trophozoites were analysed on SDS-PAGE followed by immunoblotting using anti-GFP antibody and anti-EhCS1 antibody. (D) Pseudopodal localization of EhC2B. The red square represents the magnified view. (E) Relative fluorescence intensities of EhC2B were quantified in pseudopod and non-pseudopod regions of stably-expressing trophozoites. Data Points shown in the graph represent mean, and SEM (error bars) for N = 50 cells and statistical significance is shown by ***P≤0.001. (F) The localisation of GFPEhC2B in amoebic trophozoites in the presence of BAPTA-AM or DMSO. (G) Normalised peripheral fluorescence intensities are plotted for GFPEhC2B cells in the presence of BAPTA-AM or DMSO (N = 30 cells). Statistical significance is shown by **P≤ 0.01. (H) The localisation of GFPEhC2B in amoebic trophozoites in the wild-type EhC2B strain and its calcium (D70N and D72N) and lipid mutants (K76A and N77A) cell lines. (I) Amoebic trophozoites stably expressing GFPEhC2B were incubated with Cell Tracker Orange-labelled RBCs (1:50) for 10mins at 37°C. Trophozoites were then fixed and later studied by confocal microscopy. The yellow arrow indicates the absence of GFPEhC2B at phagosome. (J) Montage of live imaging of GFPEhC2B expressing trophozoite ingesting CHO cells by trogocytosis. The time 0 min represents the initiation of the trogocytic cup. All *scale bar*, 5μm. (K) FRAP study of GFP-EhC2B, localising on phagocytic cups. Pre-bleached fluorescence intensity of GFPEhC2B from the region of interest was measured and assigned as 100%. The region of interest, as shown by the yellow circle was photobleached, and the recovery of the fluorescence intensity was monitored as described in ‘Materials and Methods.’ The corresponding magnified view of the area of interest is shown by square below (S3 Movie). (L) The recovery of fluorescence was fitted into an exponential model. Recovery curve shows the corresponding times for 50% recovery (τ_1/2_), and values are shown by Mean and SEM (error bars) for 6 independent cells. (All *scale bars*, 5μm; DIC, Differential interference contrast).

To study the cellular localisation and function of EhC2B, we cloned EhC2B into an amoebic expression plasmid, pEhEx, with an N-terminal GFP-tag fused to EhC2B. pEhEx-GFPEhC2B was stably transfected into the amoebic trophozoites. The expression and the subcellular localisation of EhC2B were determined by fluorescence imaging of paraformaldehyde-fixed trophozoites using confocal microscopy. EhC2B was found to be localised beneath the plasma membrane as well as throughout the cytosol (Fig 3A). The line intensity plots across transects of the GFPEhC2B overexpressing amoebic trophozoites further demonstrate the abundance of EhC2B underneath the plasma membrane. It should be noted that this enrichment is not dramatic, and many times, exist underneath only certain regions of the plasma membrane (Fig 3B) (S6 Fig). We also validated the expression of GFPEhC2B (49kDa) by Western Blot (Fig 3C).

EhC2B was also found to enrich the plasma membrane extensions such as pseudopods (Fig 3D). These pseudopods were of variable sizes. The normalised mean fluorescence intensities between the pseudopod and non-pseudopod regions were compared in 50 cells. In the pseudopods, the fluorescence intensity of GFPEhC2B was nearly two-fold higher than that from the non-pseudopod regions (Fig 3E). We also observed the same pattern in time-lapse images of the amoebic trophozoites from live-cell confocal microscopy (S7 Fig and S1 Movie). This montage clearly depicts the transient enrichment of GFP fluorescence underneath the plasma membrane (highlighted by arrow) along with the formation and dissolution of the pseudopod-like protrusive structures (S7 Fig). From the earlier studies, we know that localisations of most of the C2 domain-containing proteins are calcium-dependent. Our *in vitro* results had also suggested that EhC2B is a calcium-dependent phosphatidylserine-binding protein (Fig 2C and 2D). Therefore, we hypothesised that the peripheral localisation of EhC2B should depend on the intracellular calcium concentration. We used BAPTA-AM and depleted the intracellular Ca^2+^ level of the amoebic cell and studied the localisation of EhC2B using confocal microscopy. In the presence of the intracellular calcium chelator, GFPEhC2B shows total cytoplasmic localisation (Fig 3F). The effect of BAPTA-AM on EhC2B localisation was quantified in 30 cells (N = 30). The mean peripheral fluorescence intensity of the treated cells had decreased threefold, then in the case of an untreated cell (Fig 3G). The quantification was based upon the observations from three independent experiments (n = 3). These results further support the hypothesis that the peripheral localisation of EhC2B depends on calcium. In the amoebic trophozoites, both calcium-mutants, as well as lipid mutants of EhC2B, also showed the localization pattern similar to EhC2B after BAPTA treatment (Fig 3H).

We also know that *E*. *histolytica* is a professional phagocyte. It efficiently phagocytoses host erythrocytes. The RBC containing phagosomes appear rapidly after the engulfment of the cargo. To find out whether EhC2B localises on other membrane extensions such as to phagocytic cups or maybe at phagosomes, we incubated the GFPEhC2B expressing trophozoites with erythrocytes to initiate phagocytosis. As revealed by confocal microscopy, EhC2B localised at the tips of phagocytic cups. However, we could not detect it on the RBC containing phagosomes as shown by the yellow arrow (Fig 3I). This observation hinted that EhC2B might play a role during the early stages of phagocytosis. We also investigated the localisation of EhC2B in the presence of another phagocytic cargo, i.e. CHO cells via live-cell confocal microscopy. Interestingly, EhC2B-expressing amoebic trophozoites did not engulf the entire CHO cells. It ‘bites off’ the cell and then engulfs only that part of the cell (S2 Movie). This phenomenon is typically known as trogocytosis. Here also, we observed that EhC2B specifically localised more at the extending arms of the trogocytic cup (Fig 3J). We also validated our results using fluorescence recovery after photobleaching (FRAP). As depicted in Fig 3K, a specific region of the cell with the phagocytic cup was photobleached. The restoration of the fluorescence in the bleached area was measured with time. A nearby unbleached region within the same cell served as a control. The fluorescence intensity of the unbleached region indicates that the loss of fluorescence in the bleached region is specific and not an artefact of imaging. The fluorescence measurements for the test and control regions were normalised to their pre-bleach levels (defined as 100% relative intensity) (Fig 3K) (S3 Movie and S5 Movie). The mean fluorescence intensity was plotted against time to enable the statistical analysis of multiple experiments (Fig 3L). The plot represents analysis for N = 6 cells. The rapid recovery of the fluorescence was observed with a mean t_1⁄2_ of 4.44 ± 1.32s (Fig 3L).

### EhC2B promotes erythrophagocytosis in *E*. *histolytica*

**Fig 4:**
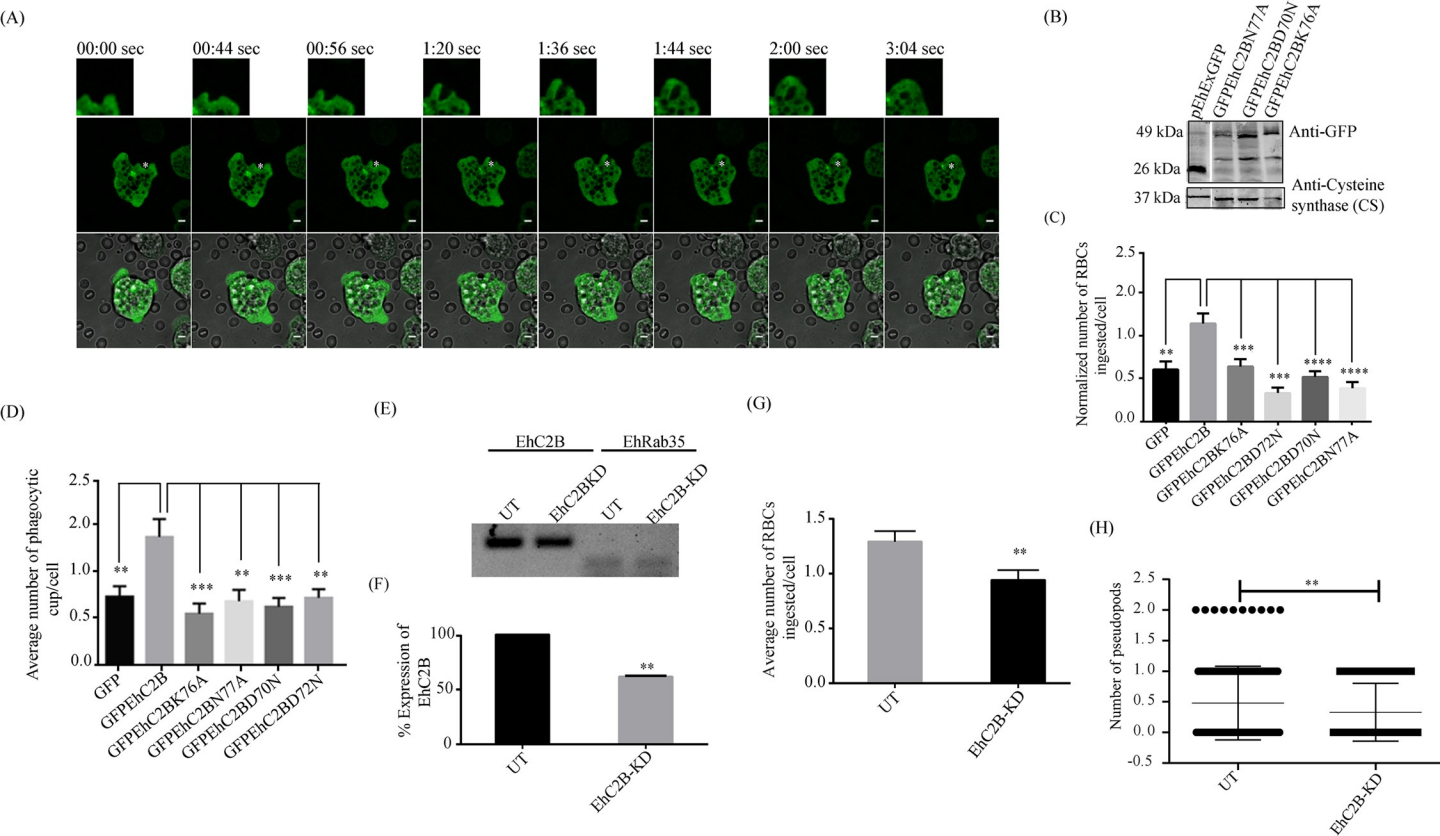
(A) Time-lapse images showing the *de novo* phagocytic cup formation during erythrophagocytosis (white asterisk) in GFPEhC2B expressing amoebic trophozoites. The montage shows the formation of the phagocytic cup in real-time. The time 0 second represents the initiation of the phagocytic cup. All *scale bar*, 5μm. (B) Total cell lysates (500μg) from GFP (vector control; 26kDa) and the lipid (N77A and K76A), as well as calcium (D70N) mutants of GFPEhC2B (49kDa), transfected amoebic trophozoites were analysed on SDS-PAGE followed by immunoblotting using an anti-GFP antibody. These blots were also probed for the loading control using an anti-EhCS1 antibody (MW-37kDa). (C) The number of RBC ingested by wild-type or mutant EhC2B expressing trophozoites transfected was quantified using confocal microscopy as mentioned in Materials and Methods' section. (D) The number of phagocytic cups present in wild-type or mutants EhC2B was enumerated and plotted. All the bar graphs represent Mean and SEM (error bars) for N = 50 independent cells. Statistical significance was determined using unpaired two-tailed Student's t-test. All significant differences are shown by *P≤0.05, **P≤ 0.01, ***P≤0.001 and ****P≤0.0001. (E) The Representative image shows mRNA expression of EhC2B, which was analysed by Semi-Quantitative RT-PCR on 1% agarose gel using EhRab35 as control. The comparison was made following PCR using the total mRNA pool of both untransfected (UT) and pKT3M-EhC2B construct transfected strain (EhC2B-KD). (F) Relative percentage Expression of EhC2B gene was analysed using ImageJ software from Semi-Quantitative PCR agarose gels of (n = 2) independent set of experiments. The statistical significance was found to be **P = 0.0099. (G) The number of RBCs ingested by untransfected or pKT3M-EhC2B transfected strain was enumerated and plotted. The bar graph represents Mean and SEM (error bars) for N = 195 independent cells from n = 3 independent experiments. The statistical difference was found to be significant at **P = 0.0119. (H) A dot-plot graph shows the number of phalloidin568 labelled actin enriched pseudopods in each cell for both untransfected (UT) as well pKT3M-EhC2B (EhC2B-KD) transfected strains of trophozoites. The Error bars represent the Mean and SEM for N = 195 independent cells from n = 3 independent experiments. The significant statistical difference is shown as **P = 0.0185.

The fixed-cell imaging revealed the localisation of EhC2B at the pseudopodal structures and advancing tips of phagocytic cups which hinted that the protein might be involved in amoebic phagocytosis (Fig 3I). We further investigated its localisation in live trophozoites in the presence of RBC. GFP-tagged EhC2B expressing trophozoites were incubated with RBCs and dynamics of EhC2B localisation was studied under physiological conditions using confocal microscopy. Live-cell imaging data revealed that upon incubation with RBC, GFPEhC2B rapidly accumulated at the site of RBC attachment (Fig 4A). EhC2B then continued to localise close to the tips of the arms of the phagocytic cups until the closure of the cups. Once the RBC is internalised, and phagosome formed, EhC2B gets detached from the membrane. Thus, the newly formed phagosome is devoid of the EhC2B. We observed this pattern consistently in multiple independent experiments (S4 Movie and S6 Movie).

Through *in vitro* liposome pelleting assay, we have already shown that mutation of the conserved calcium-binding residues or lipid-binding residues significantly affected the membrane localisation of EhC2B (Fig 2C and 2D). We employed these mutants to study whether EhC2B indeed has any role in erythrophagocytosis. We stably transfected the amoebic trophozoites with calcium (GFPEhC2BD70N and GFPEhC2BD72N) and lipid (GFPEhC2BK76A and GFPEhC2BN77A) binding mutants of EhC2B. As expected, the mutants showed mostly cytosolic localisation (Fig 3H). The amoebic expression of these mutants was also validated by Western Blot (Fig 4B). To determine the impacts of these mutations on erythrophagocytosis, we performed RBC uptake assay. Each of the wild-type EhC2B, as well as its mutant-expressing transgenic trophozoites, were allowed to internalize RBCs for 10 min at 37°C before fixation in 4% paraformaldehyde. A quantitative comparison revealed that the average number of RBCs ingested per trophozoite was significantly less for the mutant-expressing cell lines as compared to the cell line expressing wild-type EhC2B (Fig 4C). The wild-type EhC2B expressing trophozoites showed a nearly two-fold increase in RBC uptake as compared to the vector control. The quantification is based on three independent experiments, with a total of 90 cells (Fig 4C). Next, we quantified the number of phagocytic cups in the wild-type EhC2B and mutants expressing trophozoites. Transfected trophozoites were incubated with human RBCs for 5 min at 37°C, and the numbers of phagocytic cups were enumerated from confocal micrographs. The EhC2B mutants showed significantly less number of phagocytic cups compared to the wild-type protein (Fig 4D). Collectively, these data suggest that EhC2B is recruited to the plasma membrane during RBC internalisation in a calcium-dependent manner and plays an essential role during erythrophagocytosis. In the current study, we have established that EhC2B is an effector of Rab GTPase EhRab21, *in vitro* (Fig 1C–1F). It is well known that GTPase-effector interactions have implications on cellular functions of the GTPases. Therefore, in an attempt to delineate the cellular implication of EhRab21-EhC2B interaction, we assessed the possibility of EhRab21WT playing a role in erythrophagocytosis. We generated the HAEhRab21 overexpressing amoebic trophozoites by stably transfecting with pEhEx-HAEhRab21 and validated the expression of the GTPase by Western blot using an anti-HA antibody (S8A Fig). Cell tracker labelled RBCs (see methods) were used to measure the phagocytic efficiency of these trophozoites against control amoebic trophozoites stably transfected with pEhEx-HA. Amoebic cells and RBCs were incubated together at 1:50 ratio for 10 mins at 37°C. As shown in S8 Fig, we did not observe any effect of over-expression of EhRab21 on erythrophagocytosis (S8B Fig). To further validate the importance of EhC2B in erythrophagocytosis, we have generated knock-down strain using trigger-based RNAi approach for gene silencing [37]. In triggered-based gene silencing approach, we have inserted EhC2B gene in pKT3M vector (a kind gift from Dr. Upinder Singh’s lab) using SmaI and XhoI restriction sites and transfected the construct into the amoebic trophozoites to generate the knock-down strain. The knockdown efficiency of EhC2B in amoebic trophozoites was quantified by semi-quantitative RT-PCR. It shows that the expression of the pKT3M-EhC2B in amoebic trophozoites has reduced the endogenous EhC2B RNA level by nearly 40% (Fig 4E and 4F). Consequently, the phagocytosis efficiency of knock-down cells was also reduced by 30% in comparison to untransfected trophozoites (Fig 4G). Since EhC2B had shown the localisation at the pseudopods, we also quantified the effect on the number of pseudopods in the knock-down cell line. It was found that the number of pseudopods has reduced significantly in comparison to untransfected trophozoites. These results thus established that EhC2B has a role in phagocytosis as well as in the formation of pseudopods (Fig 4H).

### EhC2B is an actin nucleator

**Fig 5:**
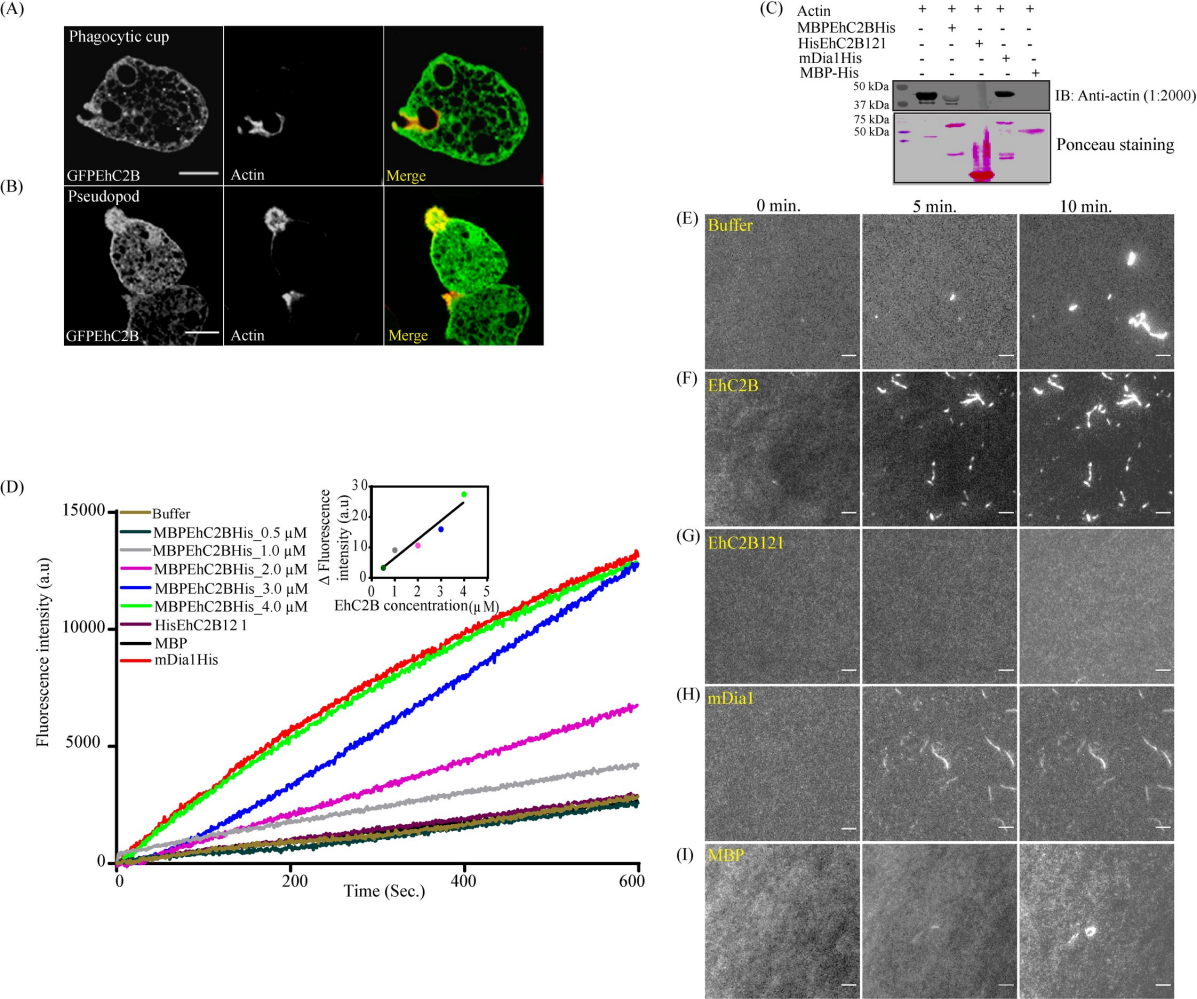
Colocalization of GFPEhC2B with actin at (A) Phagocytic cup (B) Pseudopod. The GFPEhC2B overexpressing amoebic trophozoites were immunostained with anti-actin antibody followed by Alexa568 labelled phalloidin and visualised under a confocal microscope. (C) His pull-down assay for direct interaction between EhC2B and actin. One micromolar of MBP-EhC2B, His-EhC2B121, MBP or His-mDia1 was immobilized on Nickle-NTA beads and incubated with an equimolar concentration of monomeric rabbit muscles actin at 4°C. The binding was detected by immunoblotting using an anti-actin antibody. (D) Pyrene-actin nucleation assay. 0.25μM pyrene-labelled of rabbit muscles monomeric actin was mixed with 47 μl of G buffer (10 mM Tris pH 8.0, 0.2 mM ATP, 0.2 mM DTT,0.2 mM CaCl_2_), 6μl of exchange buffer (10 mM EGTA, 1 mM MgCl_2_), and incubated for two minutes at room temperature. Finally, 5μl of ion mixture (1 M KCl, 40 mM MgCl_2_, 10 mM ATP) was added to the reaction, and actin polymerization was initiated. The fluorescence intensity of polymerization was measured for 600 seconds in the presence of a different concentration of EhC2B, EhC2B121 (C2 domain), mDia1 and MBP at 365nm excitation and 407nm emission wavelength. (E) Real-time actin polymerization using TIRF microscopy. Assembly of actin monomer was initiated from 0.1μM of rabbit muscles actin by the combination of G-buffer (10 mM Tris (pH8.0) 0.2 mM ATP, 0.2 mM DTT, 0.2 mM CaCl_2_), exchange buffer (10 mM EGTA, 1 mM MgCl_2_), and ion mix (1 M KCl, 40 mM MgCl2, 10 mM ATP) as mentioned in ‘ Material and Methods' section. The actin filaments were stained with 30nM of phalloidin labelled with Alexa-568. The polymerization of actin monomer was monitored for 30 minutes in real time under TIRF microscope (Nikon T*i*2) using 60X objective lens with 1.5X zoom (S7–S11 Movies). To detect the effect of EhC2B on actin dynamics, the actin nucleation reactions were set in the presence of 0.1 μM of (F) MBP-EhC2B (G) HisEhC2B121 (H) mDia1 (I) MBP (All s*cale bars*, 5 μm).

So far, we have observed that EhC2B localises at the phagocytic cup and pseudopods and is involved in erythrophagocytosis by the amoeba. However, the assembly of the actin monomer is essential for successful completion of the pseudopod formation as well as phagocytosis [10,38,39]. Several amoebic calcium-binding proteins are known to be involved in actin dynamics at the phagocytic cup [23,40]. Confocal microscopy of paraformaldehyde-fixed amoebic trophozoites revealed that EhC2B too, colocalises with actin at the phagocytic cup as well as pseudopods (Fig 5A and 5B). To determine whether EhC2B can directly bind to the actin, we carried out His pull-down based binding assay. MBPEhC2BHis or HisEhC2B121 was immobilised on the Ni-NTA beads as bait and incubated with the purified rabbit muscles actin protein. The beads were then washed extensively with wash buffer and subjected to SDS-PAGE followed by Western blot using an anti-actin antibody (Fig 5C). As shown in Fig 5C, while the full-length MBPEhC2BHis, fused with MBP and His tag at the N-terminal and C-terminal end, respectively binds to actin, HisEhC2B121 could not show any detectable binding. We have also used mDia1 as a positive control. It is a well-established actin nucleator [41].The results from the above assay established that EhC2B binds to actin in the amoebic trophozoites. It also suggested that EhC2B actin interaction is specifically mediated by the C-terminal proline-rich region of EhC2B (Fig 5C).

Next, to test whether the binding of EhC2B has any implication on actin dynamics, we carried out an *in vitro* actin polymerization assay, where the rise in fluorescence from pyrene-labelled actin was measured as the readout of actin polymerization. We also decided to investigate whether EhC2B has any role in the nucleation step or not. Actin nucleus is essential for the filament formation, elongation, and is considered as the rate-limiting step in actin polymerization [42]. For nucleation assay, 0.25 μM of pyrene-labelled rabbit muscles actin (RMA) was dissolved in G-buffer (10 mM Tris pH 8.0, 0.2 mM ATP, 0.2 mM DTT, and 0.2 mM CaCl_2_). The actin assembly was initiated after adding the exchange buffer (10 mM EGTA, 1 mM MgCl_2_), and ion mix (1 M KCl, 40 mM MgCl_2_, 10 mM ATP) into the reaction mixture. The pyrene fluorescence due to actin polymerization was recorded for 600 seconds at an excitation wavelength of 365nm and an emission wavelength of 407nm. A rise in the pyrene fluorescence with the addition of an increasing amount of MBPEhC2BHis suggested a possible role of the protein in actin nucleation (Fig 5D) (S9 Fig).

Finally, to validate the above results and directly observe actin nucleation in the presence of EhC2B, we used total internal reflection fluorescence microscopy (TIRFM) that allowed real-time imaging of elongating actin filaments (Fig 5E–5I) (S7–S11 Movies). The actin nucleation reaction mixture is composed of 0.1μM RMA, suspended in G buffer, supplemented with exchange buffer and ion mix. The reaction mix was transferred onto the poly-L-lysine coated glass-bottom imaging dish and then incubated at room temperature for 2 minutes. The polymerization of actin was visualised in real-time by staining the actin filament with Alexa568 labelled phalloidin. The background fluorescence from the TIRFM was estimated by allowing RMA monomer to polymerize in the absence of phalloidin. It was observed that full-length EhC2B induces more actin filaments in a given time as compared to the control (Fig 5F and S7–S11 Movies). TIRFM results also revealed that the EhC2B121 could not stimulate the formation of the actin filament, which was also inconsistent with the result of full-length EhC2B as obtained from pyrene-fluorescence assay (Fig 5G). In summary, the above results indicate that full-length EhC2B promotes actin nucleation and relies on its C-terminal proline-rich region for actin-binding and inducing nucleation.

## Discussion

*E*. *histolytica*, a professional phagocyte ingests a variety of cargoes such as gut bacteria, dead cell corpses, and RBCs. In this study, we have established the role of EhC2B in forming pseudopods and in promoting erythrophagocytosis through the nucleation of actin. EhC2B is a C2 domain-containing protein and binds to the phosphatidylserine in a calcium-dependent manner besides some other phosphoinositides. It is identified as a GTP specific interacting partner of EhRab21 from *E*. *histolytica*. Using extensive biochemical and biophysical approaches, we have ascertained it as an effector of EhRab21 *in vitro*. Also, the C2 domain of EhC2B itself is sufficient for the interaction with EhRab21. However, the physiological relevance of EhC2B-EhRab21 complex required further exploration. In an attempt to address this, EhRab21 role was studied in erythrophagocytosis. Our results (S8 Fig) suggest that EhRab21 is not involved in erythrophagocytosis, which is also consistent with the earlier observation made by Emmanuel *et al*. [30].

Till date, only very few effectors for the Ras superfamily small GTPases have been characterized in *E*. *histolytica*. EhPAK4 and EhPAK5 have been shown to be effectors for EhRacC while EhFormin was shown to be an effector for EhRho1 [11,43]. EhFormins are known to act in response to extracellular signals associated with F-actin at multiple subcellular structures, leading to cytoskeleton regulation in various cellular processes [12].

Generally, C2 superfamily members show promiscuity in terms of their preference for the lipid head groups [36]. For instance, the crystal structure of PKC alpha, a C2 domain protein in complex with Ca^2+^, PS, and PtdIns(4,5)P2 has shown that calcium is coordinated to the conserved amino acid residues in the calcium-binding pocket of the protein (PDB ID: 3GPE). It also revealed that PS interacts with the protein through the conserved calcium and lipid-binding residues [44]. Our biophysical and cellular studies have also confirmed that EhC2B preferentially binds to phosphatidylserine in Ca^2+^ dependent fashion besides showing binding to few other phosphoinositides. In amoebic trophozoites, EhC2B calcium mutants are cytosolic and additionally, showed significantly reduced phagocytic efficiency. These observations thus strengthen the importance of calcium in regulating the function of EhC2B. The involvement of some of the C2 domain proteins in phagocytosis, such as PKC2 and PI3K, have also been previously reported in the mammalian cells as well as in *E*. *histolytica* [23,45,46].

In *E*. *histolytica*, it is well documented that the initial contact of the amoebic cell with the RBCs alters the intracellular calcium levels by an unknown mechanism and induce the rapid polymerisation of the actin at the site of contact [21,24,38,47,48]. Earlier studies have revealed the role of a few CaBPs during early stages of phagocytosis. However, spatiotemporal distribution of EhC2B at the phagocytic cup is unique in comparison to them [23–25,27,28]. EhC2B is highly concentrated at the tips of the phagocytic cup and moves along with the leading ends of the cup. Once the cargo is internalised, EhC2B dissociates from the membrane; hence, it is not present on the phagosome. In the mammalian system, the cellular localisation of CDC42 and ARF6 resembles that of EhC2B during the early stage of phagocytosis [49]. Both these proteins participate in the closure of the phagocytic cup. CDC42 recruits WASP which in turn activates the Arp2/3 complex and thus regulates the advancement of the cup over the cargoes by local stimulation of the actin polymerization. ARF6 activates the PI(4)P-5-Kinase and PLD2, which regulate the membrane curvature and fusion during the closure of the cup. Similarly, the accumulation of EhC2B at the leading edge of the cup implies about its possible role during the closure of the cup.

The localisation of EhC2B at the phagocytic cup was moreover verified by fluorescence recovery after photobleaching (FRAP) based study. Quick recruitment of EhC2B at the phagocytic arms following the temporary suspension by photobleaching directly hints at the requirement of EhC2B for the phagocytic cup formation (Fig 3K and 3L). Previously, a similar study was performed to show the recruitment of the EhRab35 at the cytosolic vacuoles [50]. Interestingly, EhRab35 also localizes to phagocytic cup and is involved in erythrophagocytosis by the amoeba. However, the localisation for EhRab35 differs from that of EhC2B, mainly in two aspects. First, unlike EhC2B, EhRab35 is present throughout the arms of the phagocytic cup. Moreover, post enclosure of the phagocytic cup, EhRab35 continues to localize on the phagosomal membrane and plays an essential role in the biogenesis of the phagolysosome in the parasite [50].

Regulated dynamics of the actin cytoskeleton is indispensable for the amoebic pathogenesis. It was shown that each step of the RBC uptake such as adhesion, cytolysis, and phagocytosis is cytochalasin dependent which indicates the importance of actin dynamics in amoebic phagocytosis [38]. Many actin regulatory/binding proteins govern the constant turnover of the actin filament [51]. In *E*. *histolytica*, several actin-binding proteins (ABPs) for example, EhFormin1, EhCaBP1, EhCaBP3, EhCoactosin, and EhNCABP116 have already been characterized and are shown to be involved in phagocytosis [10,12,23,27,52,53]. Ca^2+^ is another prominent regulator of the actin cytoskeleton in *E*. *histolytica* [54,55]. The events describing the RBC phagocytosis by the amoebic trophozoites are (a) upon initiation of the phagocytosis, EhC2PK is recruited at the site of RBC attachment in a calcium-dependent manner. (b) EhC2PK then binds to EhCaBP1 in a Ca^2+^ independent manner. (c) EhCaBP1 recruits EhAK1 (Ca^2+^ dependent) followed by Arp2/3 complex via EhARPC1. EhARPC2 recruits EhCaBP3 and EhMyosin 1B to the phagocytic cups in the presence of calcium. Other actin modulating molecules are also recruited at this stage. (d) Progression of phagocytic cups, activation of actin dynamics pathway by recruitment of Arp2/3 complex, and efficient actin polymerisation by phosphorylation of monomeric actin by EhAK1. (e) Finally, the closure of the cup and formation of mature phagosome requires EhCaBP3, EhMyosin 1B, EhAK1, Arp2/3 complex (EhARPC1 and EhARPC2 and several amoebic Rab GTPase [24,50,56–58]. Thus, the calcium-dependent concerted activities of these proteins facilitate regulation of amoebic cytoskeleton dynamics, consequently leading to phagocytic cup formation and closure. The current study demonstrates that EhC2B can directly associate with the plasma membrane through its C2 domain and modulate actin cytoskeleton, during erythrophagocytosis. It is interesting to note that the localization of EhC2B resembles that of EhAK1 and EhARPC1 both of which are recruited by EhCaBP1 and involved in actin polymerization during phagocytosis [59,60]. It is not clear from our results whether EhC2B could be part of the above network of amoebic proteins or if it functions as an independent machinery for actin nucleation during phagocytosis. Further proteomics-based studies should be sought to reveal its participation in the larger network of calcium binding proteins involved in actin dynamics during the process.

Through our spectroscopic and high-resolution microscopy studies, we demonstrated that full-length EhC2B, a calcium-binding C2 domain protein, is an actin nucleator. However, the C2 domain of EhC2B alone does not bind to actin monomer, nor does it stimulate the actin nucleation *in vitro* (Fig 5D and 5E–5I). Thus, our results suggest that the actin nucleation is mediated by the C-terminal proline-rich region of the protein. The comparison of the nucleation activity with human mDia1 further confirmed EhC2B to be functionally active actin nucleator where mDia1 belongs to the formin family of actin nucleators [41,61]. The formin family of proteins contain a polyproline region which is known to interact with profilin to recruit the actin monomer [13,14]. In *E*. *histolytica*, several formin proteins (EhFormin1-8) were identified. Among them, EhFormin 1 and 2 were shown to be involved in cell division and proliferation. Although the authors have shown that EhFormin 1 and 2 bind to the F-actin, their role in actin nucleation was not explored in the study [12]. Therefore, to best of our knowledge EhC2B is the first actin nucleator identified from *E*. *histolytica*.

**Fig 6:**
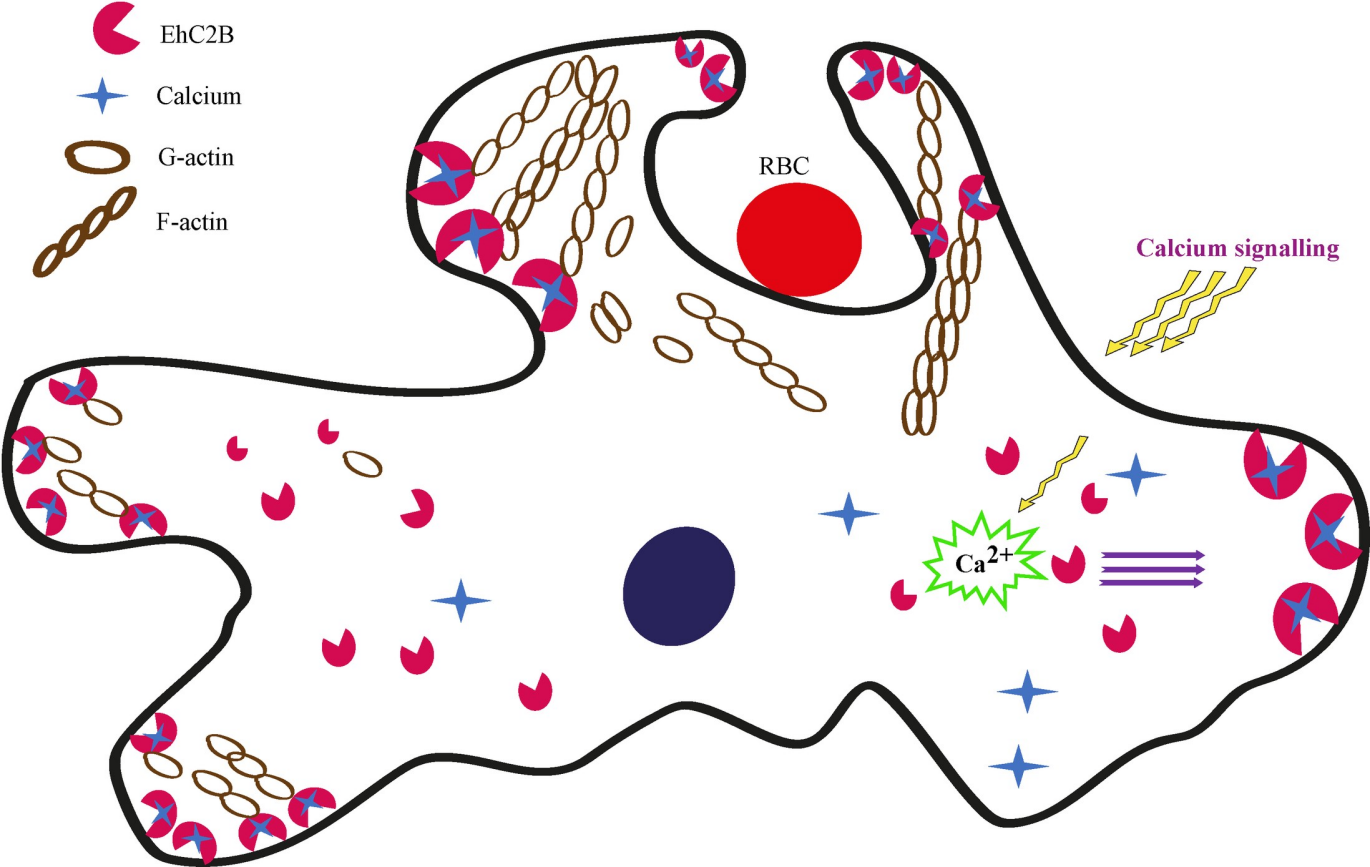
EhC2B regulates the formation of membrane pseudopods and phagocytic cups by nucleating actin in *E*. *histolytica*. In the presence of calcium, EhC2B relocates transiently. It binds to variants of phospholipids in the inner leaflet of the plasma membrane through some of the conserved residues in its N-terminal C2 domain. The C-terminal proline-rich region of EhC2B then binds to the G-actin monomer and causes actin nucleation, thereby, inducing actin polymerization just beneath the plasma membrane. This nucleating activity of EhC2B thus leads to the formation of protrusive membrane structures such as pseudopods or phagocytic cups in the presence of cargo such as RBCs.

Thus collectively, we hypothesise that under the influence of calcium signalling, EhC2B mobilise and binds to variants of phospholipids in the inner leaflet of the plasma membrane through its N-terminal C2 domain. The C-terminal proline-rich region of EhC2B then binds to the actin and following actin nucleation, induce its polymerization just beneath the plasma membrane (Fig 6). Thus, EhC2B could potentially generate a driving force to push the plasma membrane to form membrane protrusions known as pseudopods. These pseudopods might have a role in amoebic migration on the substratum. Similar membrane deformation occurs during the biogenesis of phagocytic cups, although biochemically they are known to be distinct [15,62].

In summary, this study demonstrates that EhC2B interacts with EhRab21 in a nucleotide-dependent fashion. Furthermore, EhC2B is a calcium-dependent phosphatidylserine binding protein which functions as an actin nucleator during the early stage of erythrophagocytosis and thereby contributing to the pathogenesis and survival of the amoeba.

## Materials and methods

### Recombinant protein expression and purification

The His-tagged C2 domain of wild-type EhC2B (His-EhC2B121) (*E*.*coli* codon-optimized, Mutagenex Inc.), calcium-binding mutants of EhC2B (D18N, D72N) and lipid-binding mutants of EhC2B (K76A, N77A, K29A/K76A/N77A), as well as MBPEhC2BHis (cloned in pETMM42 vector), were expressed and purified from E. coli BL21(DE3*)* cells. The cultures were induced at the OD_600_ 0.6 with 0.5 mM isopropyl 1-thio-β-D-glucopyranoside and allowed to grow for overnight at 20°C. Cells were grown, and harvested by centrifugation (3000 x g, 15 min, and 4°C). The cell pellet was resuspended in lysis buffer [(50 mM Tris (pH 8.0), 300 mM NaCl, 50μg/ml of benzamidine, 2 mM β-mercaptoethanol)]. The cells were lysed, and the lysate was clarified by centrifugation at 50,000 × g for 30 min at 4°C. Proteins were purified using affinity chromatography, followed by size exclusion chromatography (GE Healthcare).

### GST/His pull-down assay

One micromolar of GDP/GppNHp bound GSTEhRab21protein was allowed to bind with the sepharose beads and incubated with 5 μM MBPEhC2BHis or HisEhC2B121 for 1 hour at 4°C on rotamer in binding buffer (25 mM HEPES, pH 7.2, 100 mM NaCl, and 2 mM DTT) containing an excess of nucleotide. The binding of GSTEhRab21to HisEhC2B121 or MBPEhC2BHis was detected by immunoblotting using anti-His and anti-MBP antibody, respectively. Similarly, to detect the actin-binding, one micromolar of MBPEhC2BHis and HisEhC2B121 protein was allowed to bind with the Ni-NTA bead and incubated with 1μM of purified rabbit muscles actin (Thermo scientific) for one hour. The binding was detected by immunoblotting using an anti-actin antibody.

### Fluorescence measurements of effector binding

Fluorescence measurements were performed with a fluoromax-4 spectrofluorometer (Horiba Jobin). Fluorescence of MantGppNHp (Sigma-Aldrich) was measured directly at 360nm excitation and 440nm emission wavelength. Experiments were performed in a buffer containing 25 mM HEPES (pH 7.3), 100 mM NaCl, and 2 mM DTT at 25°C. EhRab21 was loaded with MantGppNHp, GDP and GppNHp and passed through NAP-5 column to remove unbound nucleotide. All the reactions were performed separately in a cuvette containing 100μl reaction volume, and the fluorescence intensity of each scan was recorded and overlaid.

### Isothermal titration assay

ITC experiments were carried out to determine the thermodynamic parameters for interaction between Ca^2+^ and EhC2B121/EhC2A or EhRab21 and full-length of EhC2B or its C2 domain (EhC2B121). Proteins were dissolved in 25 mM HEPES (pH7.4), 100 mM NaCl, 1 mM DTT and 5% glycerol. 76 μM of EhRab21 solution was titrated into 10 μM of HisEhC2B121 or MBPEhC2B in the presence of GDP and GppNHp at 25°C. Similarly, 700μM Ca^2+^ was titrated into 50 μM of wild-type and calcium mutant D72N, of EhC2B121. All the experiments were performed using Nano ITC (TA Instruments). Data were processed using nano analyze software to extract the thermodynamic parameters ΔH, Kd (1/Ka) and the stoichiometry (n), ΔG and ΔS were derived from the relationship: ΔG = -RTlnKa and ΔG = ΔH—TΔS.

### Lipid overlay assay

Lipid overlay assay was performed using PIP strips according to the manufacturer's instructions (Thermo Scientific). The membrane was incubated with 0.5 mg/ml EhC2B for 4 hours in blocking buffer at room temperature. The HisEhC2B121 protein bound to the lipids was detected by anti-His antibody [1:10000 (Thermo Scientific)].

### Liposome preparation

All the phosphoinositides were protonated before usage. Briefly, powdered lipids were resuspended in chloroform (CHCl_3_), dried under argon. The remaining moisture was removed by keeping the lipid in a desiccator for 1 hour followed by resuspending it into a mixture of CHCl_3_: Methanol (MeOH): 1N hydrochloric acid in a 2:1:0.01 molar ratio. The lipids were dried once again and allowed to desiccate followed by resuspending in CHCl_3_: MeOH in a 3:1 ratio and dried again under argon. The dried lipids were resuspended in CHCl_3_ and stored at -20°C. The control liposomes were prepared by mixing POPC and POPE in a 90:10 molar ratio. The phosphoinositides containing liposomes were prepared by mixing POPC, POPE and PIPs in an 80:10:10 molar ratio respectively. 30% of POP was used to prepare the control, as well as phosphoinositides containing liposome. A suspension of multilamellar liposomes containing sucrose was prepared by hydrating the dried lipid in 25 mM HEPES (pH 7.2) containing 220 mM sucrose. This solution was then freeze-thawed to produce unilamellar liposomes. Liposomes were diluted 1:5 in 25 mM HEPES (pH 7.2), and 125 mM NaCl solution. The solution was centrifuged at 250,000 g to remove sucrose from the medium. The pelleted liposomes were resuspended in a solution containing 25 mM HEPES (pH 7.2), and 125 mM NaCl to the desired concentration of 0.5 mM. All liposomes were used within one day of preparation.

### Liposome pelleting

Twenty micromolars of the protein of interest wild-type, calcium (D18N, D72N) and lipid (K76A, N77A, K29A/K76A/N77A) mutants of HisEhC2B121 was added to a final volume of 100 μl of the liposome solution both in the presence and absence of Ca^2+^. The solution was incubated for 25 minutes at the room temperature to allow the interaction between EhC2B and liposomes. The solution was centrifuged at a speed of 400,000 g for 30 min and fractionated into supernatant and pellet. The pellet was resuspended in 25 mM HEPES (pH 7.2) containing 125 mM NaCl. The samples were then collected for analysis on a precast 4–12% bis-tris gel (Novex) by Coomassie staining.

### Actin nucleation assay

Pyrene-labelled rabbit muscles actin was thawed on the ice for overnight, diluted with the G buffer [(10 mM Tris (pH8.0) 0.2 mM ATP, 0.2 mM DTT, 0.2 mM CaCl_2_)] and subjected to high-speed centrifuge at 90,000 rpm for 40 minutes. The upper part of the solution was taken carefully into a separate tube without touching the bottom of the tube.

To observe the fluorescence of actin polymerization, 100μl reaction was prepared by adding mix of 47 μl G buffer, 6μl exchange buffer (10 mM EGTA, 1 mM MgCl_2_), and 0.25 μM actin mixture and then waited for two minutes. Finally, 5 μl of ion mixture (1 M KCl, 40 mM MgCl_2_, and 10 mM ATP) was added to the mix, and the total volume was made up to 100 μl with protein buffer. The reaction was transferred to the cuvette immediately after the preparation, and fluorescence intensity was measured at 365nm excitation and 407nm emission wavelength (Horiba, Scientific).

### TIRF microscopy

The reaction contains 0.1 μM rabbit muscles actin with and without EhC2B protein. The protein mixture was diluted by adding 372 μl of G-buffer, 30μl exchange buffer, 23 μl of ion mix and the total volume was maintained up to 450 μl by adding protein buffer. The composition of each buffer has been mentioned previously. The actin was stained by adding 30nM of phalloidin, and the reaction mixture was transferred to the poly-L-lysine coated glass-bottom imaging dish. The images were captured at one-minute intervals on an objective-based TIRF microscope (Nikon T*i*2) using 60X objective lens and with1.5X zoom.

### Cloning and plasmid construction

For cell-based studies, the sequence of EhC2B (EHI_059860) of *E*. *histolytica* strain HM1: IMSS was picked up from amoebadb.org. The gene was amplified by PCR using the cDNA pool with the help of Forward (5'-CCCGGGATGATCAAGATTGAATTAAAGATAATTGAAGCT-3') and reverse (5'-CTCGAGTTAGAATGGGTTGTAGTAAGGTGGTGGACATCC-3') primers. The amplified PCR product was subjected to restriction digestion with SmaI and XhoI and ligated into pEhEx/GFP using T4 ligase (New England Biolabs). For the Knockdown construct, we used pKT3M vector (a kind gift from Dr. Upinder Singh’s lab). In this construct, we had inserted the EhC2B gene into the restriction sites (SmaI and XhoI) provided [37].

### Culture of *E*. *histolytica* trophozoites

*E*. *histolytica* strain HM1: IMSS trophozoites were grown axenically at 35.5°C in a BI-S-33 medium which has been supplemented with heat-inactivated 15% (v/v) adult bovine serum (Cat. RM9981, HiMedia, India), with a mix of 100U of Penicillin/ml and 100mg Streptomycin sulphate/ml (Life Technologies, CA, USA).

### Overexpression of EhC2B and its mutant in *E*. *histolytica*

To overexpress GFP-tagged EhC2B or its mutants in *E*. *histolytica*, GFP-fusion proteins of the full-length EhC2B or its mutant genes were transfected in *E*. *histolytica* by electroporation as described previously. Briefly, trophozoites were collected from the log phase cultures and washed with 1XPBS followed by incomplete cytomix buffer (10 mM K_2_HPO_4_/KH_2_PO_4_, pH7.6), 120 mM KCl, 0.15 mM CaCl_2_, and 25 mM HEPES (pH 7.4), 2 mM EGTA, and 5 mM MgCl_2_). The washed cells were then resuspended in 0.4ml of complete cytomix buffer (incomplete cytomix containing 4 mM adenosine triphosphate and 10 mM reduced glutathione). 100 μg of plasmid DNA was added to the Cells-buffer mix and subjected to consecutive pulses of 500V voltage and 500μF capacitance (Bio-Rad Gene Pulser M Xcell) to facilitate DNA uptake by trophozoites. Electroporated trophozoites were then transferred into a drug-free medium for 48hrs at 35.5°C. Subsequently, stable clones were selected in the presence of 4μg/ml G418 (catalogue number 1720, Sigma- Aldrich). All the experiments were performed at 20μg/ml G418. Overexpression of the respective proteins was confirmed by Western blotting analysis using the indicated antibodies and by confocal microscopy.

### Western blotting

*E*. *histolytica* trophozoites were solubilised with lysis buffer containing 50 mM Tris-Cl pH 7.5, 150 mM NaCl, 1mM PMSF, 1mM β-ME and 1% NP-40 in the presence of protease inhibitor cocktail (Cat. P8340, Sigma Aldrich, USA). Proteins were resolved at 100 V on 12% polyacrylamide gels under reducing conditions. Proteins were then transferred electrophoretically onto nitrocellulose membranes. The blots were incubated with 5% BSA (Bovine Serum Albumin) as blocking buffer for an hour at room temperature and probed with mouse polyclonal anti-GFP [(1:500 dilution (Roche)] and with rabbit polyclonal anti-Cysteine Synthase (1:1000 dilution) wherever necessary. Further, it was washed and incubated with HRP-conjugated anti-mouse (1:10,000) or anti-rabbit (1:10,000) respectively and eventually developed by enhanced chemiluminescence.

### Fluorescence confocal microscopy

*E*. *histolytica* grown in glass culture tubes were chilled on ice for 10 min. Trophozoites were pelleted down at 900xg for 3mins, counted by haemocytometer, resuspended in BI-S-33 medium and transferred into 8-well chamber slides (Cat. 30108, SPL Life Sciences, Singapore) at 37°C having 5x10^4^ cells/well. After 15 mins, cells were fixed with 4% paraformaldehyde (PFA) and permeabilized with 0.1% Triton X-100 in 1XPBS. Trophozoites were then blocked using 5% foetal bovine serum in 1XPBS for 45 mins. Next, trophozoites were co-incubated with Alexa-conjugated Phalloidin568 (Life Technologies, CA, USA) secondary antibody (1:50 dilution) and DAPI (40, 6-diamidino-2-phenylindole) for 1 hr at room temperature. Following three washes with 1XPBS solution, coverslips were mounted on the glass slide using Mowiol. Slides were then examined using LSM-780 laser scanning confocal microscope (Carl Zeiss, GmbH, Jena, Germany) with a 63X/1.4 NA oil immersion objective lens. Fluorescence signals were captured in individual face (XY axes) planes throughout the cellular z-axis at 1μm intervals.

### Quantification of pseudopod numbers

The gene knock-down pKT3M vector carrying the EhC2B gene was transfected into the trophozoites. These transfected cells, as well as untransfected trophozoites, were incubated in glass wells for 10 mins and then fixed by 4% PFA. These trophozoites were then co-incubated with Alexa-conjugated Phalloidin568 (Life Technologies, CA, USA) secondary antibody (1:50 dilution). Following confocal microscopy, the numbers of the cellular protrusions filled with Phalloidin568 labelled-actin were then quantified in each cell. The analysis was performed for n = 195 cells from N = 4 set of experiments inclusive of both technical as well as biological repeats.

### RBC labelling

Five millilitres of human blood was collected to extract RBCs which were then briefly washed with 1% bovine serum albumin (BSA) (in 1XPBS) and immediately incubated with Cell Tracker Orange (50 μg/ml) (catalogue number C2927, Life Technologies) for 45 min at 37°C in the dark. Afterwards, the RBCs were subsequently washed four times with 1% BSA and then resuspended in the BI-S-33 medium for experiments.

### Erythrophagocytosis

*E*. *histolytica* trophozoites and Cell-Tracker Orange labelled RBCs were resuspended separately in incomplete BI-S-33 medium. Equal numbers of amoebic cells were then incubated with RBCs in 1:50 ratio or 1:100 ratio for assays involving EhC2B-knockdown strain, into 8-well chamber, slides for 10 min at 37°C. Cells were then fixed with 4% PFA and further processed for fluorescence microscopy.

### Live cell imaging

The trophozoites expressing GFP-tagged fusion proteins were grown in 20μg/ml G418, harvested in log phase, washed, and resuspended in BI-S-33 medium. Trophozoites were transferred to glass-bottom dishes (MatTekCorp.), allowed to settle for 10 min, and observed using an Olympus inverted confocal microscope equipped with a 60X/1.4NA oil immersion objective inverted microscope. Mercury Arc laser (488nm) was used for excitation of GFP. At least 10 examples were observed in each experiment, and one representative set of images are shown.

### FRAP analysis

FRAP was performed using the FRAP module on Olympus Confocal microscope Software. For FRAP, the amoebic trophozoites harbouring GFPEhC2Bwere observed 48 hours after addition of 20 μg/ml G418 using Olympus inverted confocal microscope equipped with 60x/1.4NA oil immersion objective and a 488 nm Mercury Arc laser. Bleaching was performed during fly forward using ROI scan features and high laser power. In the FRAP experiments, spherical areas of approximately 5 μm diameter were photobleached for 500msec, and subsequently, images of the area were collected approximately every 4 frames/s. To calculate the time of 50% signal recovery, τ_1⁄2_, the FRAP curve from each of the experiments was normalised and fitted to an exponential recovery curve. The curve fit was done with help from CellSens software for the Olympus microscope.

### BAPTA-AM treatment of amoebic trophozoites

Trophozoites were washed twice with BI-S-33 media followed by resuspension in Complete BI-S-33 media (Incomplete BI-S-33 media supplemented with 15% Albumin Bovine Serum). BAPTA-AM (dissolved in DMSO) was also added and raised to respective final working concentrations (0μM-500μM). These cells were immediately incubated into 8-well plates for 30 mins at 37°C. Afterwards, the cells were quickly fixed with 4% paraformaldehyde and processed further for fluorescence analysis.

### Statistical analysis

Statistical significance was determined by comparing the differences between different conditions and involving the use of unpaired two-tailed Student's t-test with the help of GraphPad Prism version 6.0.

## Supporting information

S1 Fig (A) MBPEhC2BHis full-length protein purification. The SDS-PAGE analysis of MBPEhC2BHis purification using Ni-NTA based affinity chromatography. (B) Size-exclusion chromatography of MBPEhC2BHis. The fraction from the affinity purification was subjected to superdex 200 prep grade column (16/60) (GE healthcare). The recombinant MBPEhC2BHis was eluted at the elution volume of 76 ml. (C) SDS-PAGE analysis of the fractions from the size-exclusion chromatography.
(TIF)

S2 Fig (A) The SDS-PAGE analysis of HisEhC2B121, purified using Ni-NTA affinity chromatography. (B) The protein bound to the Ni-NTA beads was eluted, concentrated and subjected to size exclusion chromatography using superdex75 prep grade column (16/60) (GE healthcare). The HisEhC2B121 was eluted at an elution volume of 85 ml, and the protein present in different fractions was detected by Coomassie staining.
(TIF)

S3 Fig (A) Fluorescence-based assay for EhRab21 and EhC2B binding. 1μM of Mant-GppNHp bound EhRab21 was incubated with HisEhC2B121 in the cuvette. The arrows indicate different time points where the latter protein was added to the final concentration of 2μM, 4μm, 6μM and 8 μM, respectively. The fluorescence emission intensity was measured at 360nm excitation and 440nm emission wavelength. To assess the nucleotide specific interaction of EhC2B with EhRab21, GppNHp-GSTEhRab21 and GDP-EhRab21 were added at the indicated time points to the final concentration of 20μM in the reaction mixture. (B) The overlay of the fluorescence emission spectrum of mantGppNHp-GSTEhRab21 obtained with 2μM (black), 4μM (violet), (green) and 8μM (maroon) final concentrations of the C2 domain of EhC2B. The spectrum was obtained with 20μM of GppNHp-EhRab21 (brown) and GDP-EhRab21 (red) in the reaction mixture, respectively.
(TIF)

S4 Fig Interaction of the C2 domain of EhC2A with calcium. The top panel shows the raw data obtained from the titration of 200μM of HisEhC2A (C2 domain) against 2 mM CaCl_2_ at room temperature in the ITC cell. The lower panel represents the best least-square fit of the integrated heat obtained after subtracting the heat of dilution.
(TIF)

S5 Fig Lipid overlay assay. Nitrocellulose membrane with different lipids spots was blocked by 5% BSA prepared in TBST and then incubated with (A) wild-type and (B) lipid-mutants HisEhC2B121 for 4 hours at room temperature. The binding of the C2 domain protein with lipids was detected by immunoblotting by using an anti-His antibody.
(TIF)

S6 Fig Line intensity plot of the GFPEhC2B localisation in the amoebic cell (S6A-S6D). The fluorescence intensity of GFPEhC2B was analysed by drawing a line across multiple amoebic trophozoites and change in the fluorescence intensity with the distance is shown by line intensity plot.
(TIF)

S7 Fig Times-lapse images of GFPEhC2B overexpressing amoebic trophozoites from live-cell confocal microscopy. The montage shows the abundance of GFPEhC2B beneath the membrane as indicated by white colour arrows.
(TIF)

S8 Fig Erythrophagocytosis by EhRab21. (A) Total cell lysates (100μg) from pEhEx-HA (Vector control) and pEhEX-HAEhRab21 transfected amoebic trophozoites were analysed on SDS-PAGE followed by immunoblotting using anti-HA (1:1000) antibody. For loading control anti-EhCS1 (1:1000) antibody was used. The blot was then probed with secondary antibodies, anti-mouse (1:10000) and anti-rabbit (1:1000) respectively. (B) Amoebic trophozoites were stably expressing pEhEx-HAEhRab21were incubated with Cell Tracker Orange-labelled RBCs (1:50) for 10 mins at 37°C. Trophozoites were then fixed, permeabilised, stained and later studied by confocal microscopy. The RBCs ingested per cell were enumerated manually for n = 48 cells from N = 4 set of biological repeats. The data represents Mean±SEM; ns-non significant.
(TIF)

S9 Fig Pyrene-Fluorescence based actin nucleation by EhC2B. Pyrene labelled monomeric rabbit muscles actin (0.250 μM) was allowed to polymerize in the presence of different concentrations of EhC2B or EhC2B121, mDia1, and MBP as mentioned in detail in “Materials and methods” section. The fluorescence intensity of polymerization was measured at 360 and 407nm of excitation or emission spectra, respectively.
(TIF)

S1 Movie The trophozoites expressing GFPEhC2B proteins were harvested, washed in BI-S-33 medium and transferred to glass-bottom dishes (MatTekCorp.). These trophozoites were then observed using an Olympus inverted confocal microscope equipped with 100x/1.4NA oil immersion objective. The scale bar represents 5μm. The arrows have been marked to highlight the transient enrichment of GFPEhC2B underneath membranes and pseudopod-like extensions over time.
(AVI)

S2 Movie CHO cells trogocytosis was observed for pEhEx-GFPEhC2B overexpressing amoebic trophozoites by Live Cell imaging using an Olympus inverted confocal microscope equipped with 60x/1.4NA oil immersion objective. (MP4)

S3 Movie The amoebic trophozoites harbouring GFPEhC2B were observed using an Olympus inverted confocal microscope equipped with 60x/1.4NA oil immersion objective. In the FRAP experiments, an area of approximately 5μm diameter was photobleached for 500ms, and subsequently, images of the bleached area were collected approximately every 4 frames/s.
(MP4)

S4 Movie The trophozoites expressing GFPEhC2B proteins were harvested, washed in BI-S-33 medium and transferred to glass-bottom dishes (MatTekCorp.). The trophozoites were then incubated with 1XPBS washed RBCs for 2 minutes, and immediately observed using an Olympus inverted confocal microscope equipped with a 60X/1.4NA oil immersion objective inverted microscope.
(MP4)

S5 Movie FRAP study of GFPEhC2B overexpressing trophozoites. For performing FRAP, an area of approximately 5μm diameter was photobleached for 500ms, and subsequently, images of the bleached area were collected approximately every 4 frames/s. (MP4)

S6 Movie GFPEhC2B plays role in Erythrophagocytosis. GFPEhC2B overexpressing trophozoites were incubated with RBCs for 2 minutes and and immediately imaged by continuous scan using an Olympus inverted confocal microscope equipped with a 60X/1.4NA oil immersion objective inverted microscope. (MP4)

S7 Movie Actin polymerization was acquired in the presence of buffer (Fig 5E) using an objective-based TIRF microscopy as discussed previously in “Material and Methods” section. (MP4)

S8 Movie Actin polymerization was acquired using an objective-based TIRF microscopy in the presence of 0.1μM of MBPEhC2BHis (Fig 5F) as discussed previously in “Material and Methods” section. (MP4)

S9 Movie Actin polymerization studies were performed under an objective-based TIRF microscopy in the presence of 0.1μM of HisEhC2B121 (Fig 5G) as discussed previously in “Material and Methods” section. (MP4)

S10 Movie Actin polymerization was carried out under an objective-based TIRF microscopy in the presence of mDia1 (Fig 5H) as discussed previously in “Material and Methods” section. (MP4)

S11 Movie Actin polymerization was acquired using an objective-based TIRF microscopy in the presence of MBP (Fig 5I) as mentioned in detail in “Material and Methods” section. (MP4)
