# Supplementary figures and images for: EhC2B, a C2 domain-containing protein, promotes erythrophagocytosis in Entamoeba histolytica via actin nucleation
Source: PLoS Pathog. 2020 May 4;16(5):e1008489. doi: 10.1371/journal.ppat.1008489 (PMC7197785; doi:10.1371/journal.ppat.1008489)

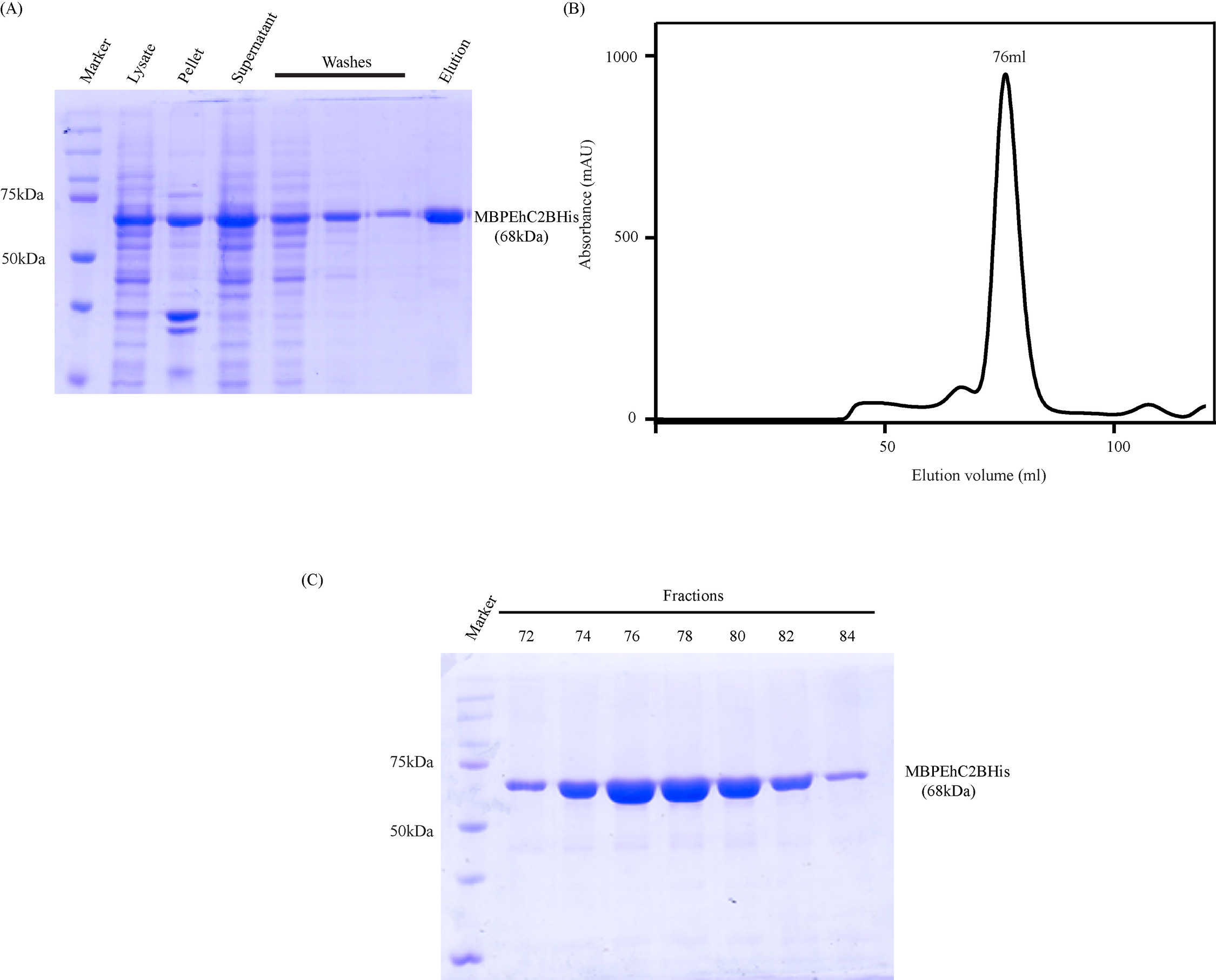

Supplement: S1 Fig — (A) MBPEhC2BHis full-length protein purification. The SDS-PAGE analysis of MBPEhC2BHis purification using Ni-NTA based affinity chromatography. (B) Size-exclusion chromatography of MBPEhC2BHis. The fraction from the affinity purification was subjected to superdex 200 prep grade column (16/60) (GE healthcare). The recombinant MBPEhC2BHis was eluted at the elution volume of 76 ml. (C) SDS-PAGE analysis of the fractions from the size-exclusion chromatography. (TIF) [file ppat.1008489.s001.tif]

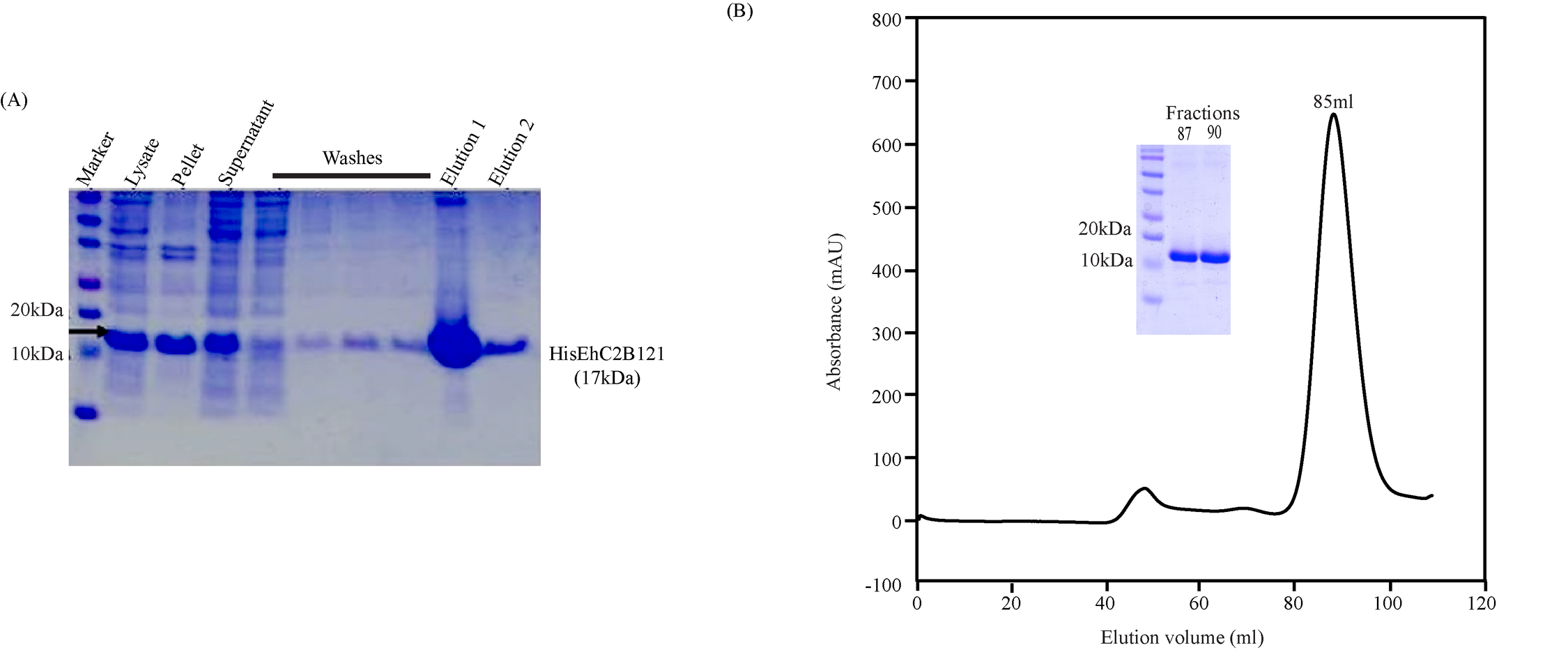

Supplement: S2 Fig — (A) The SDS-PAGE analysis of HisEhC2B121, purified using Ni-NTA affinity chromatography. (B) The protein bound to the Ni-NTA beads was eluted, concentrated and subjected to size exclusion chromatography using superdex75 prep grade column (16/60) (GE healthcare). The HisEhC2B121 was eluted at an elution volume of 85 ml, and the protein present in different fractions was detected by Coomassie staining. (TIF) [file ppat.1008489.s002.tif]

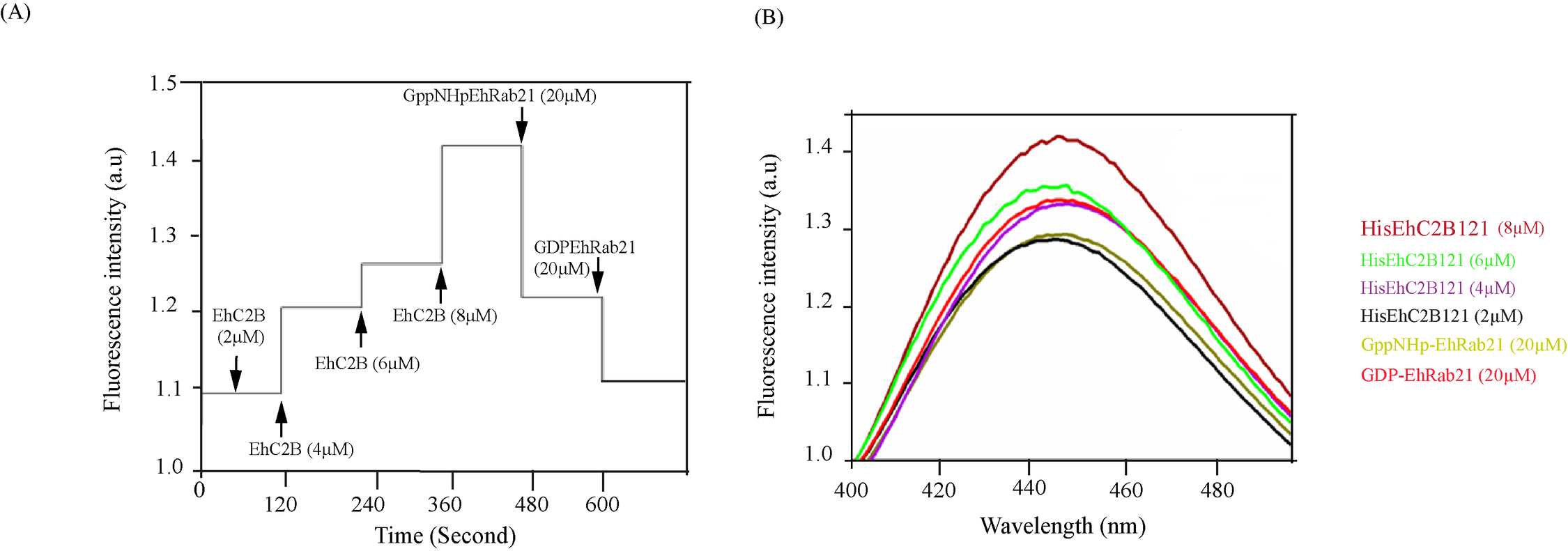

Supplement: S3 Fig — (A) Fluorescence-based assay for EhRab21 and EhC2B binding. 1μM of Mant-GppNHp bound EhRab21 was incubated with HisEhC2B121 in the cuvette. The arrows indicate different time points where the latter protein was added to the final concentration of 2μM, 4μm, 6μM and 8 μM, respectively. The fluorescence emission intensity was measured at 360nm excitation and 440nm emission wavelength. To assess the nucleotide specific interaction of EhC2B with EhRab21, GppNHp-GSTEhRab21 and GDP-EhRab21 were added at the indicated time points to the final concentration of 20μM in the reaction mixture. (B) The overlay of the fluorescence emission spectrum of mantGppNHp-GSTEhRab21 obtained with 2μM (black), 4μM (violet), (green) and 8μM (maroon) final concentrations of the C2 domain of EhC2B. The spectrum was obtained with 20μM of GppNHp-EhRab21 (brown) and GDP-EhRab21 (red) in the reaction mixture, respectively. (TIF) [file ppat.1008489.s003.tif]

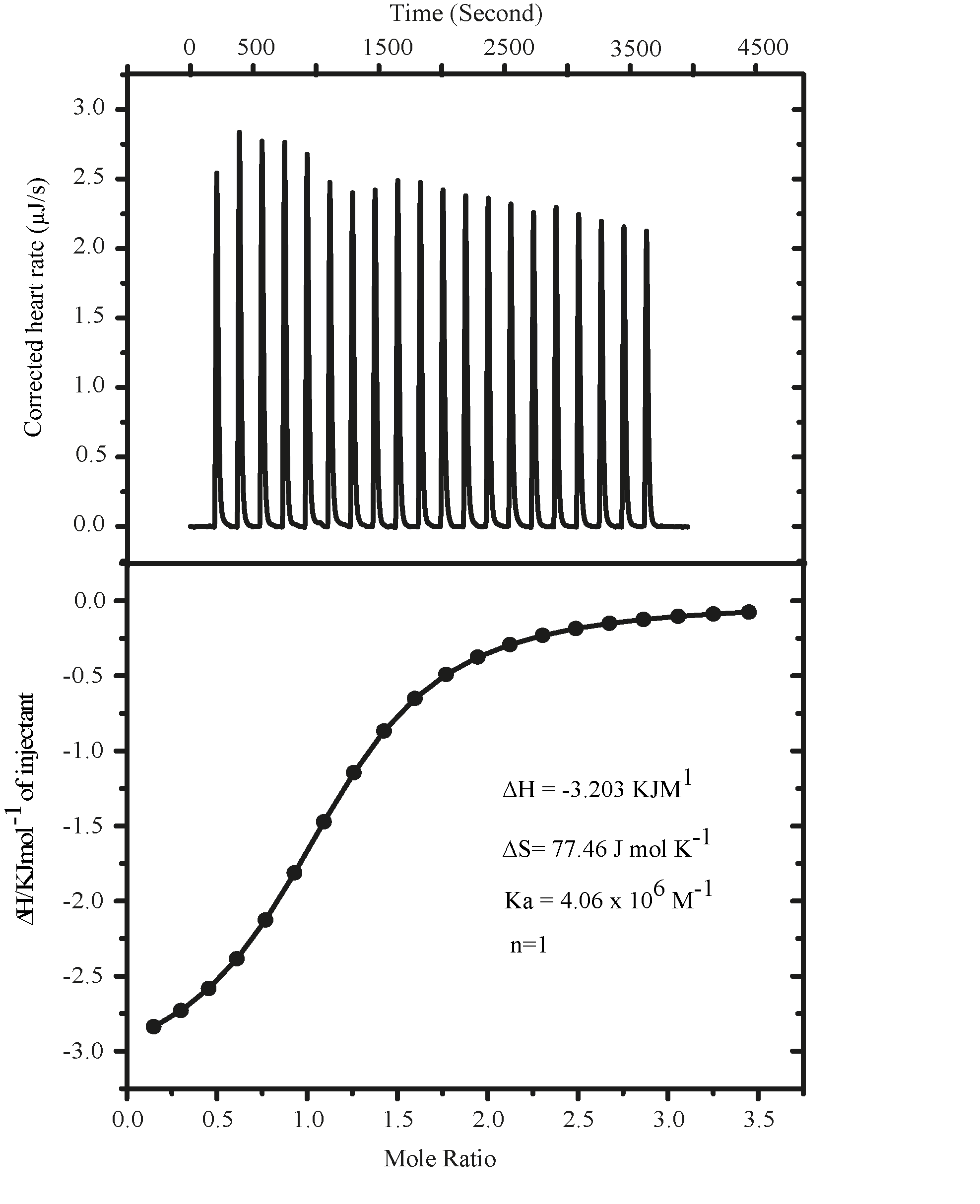

Supplement: S4 Fig — The top panel shows the raw data obtained from the titration of 200μM of HisEhC2A (C2 domain) against 2 mM CaCl2 at room temperature in the ITC cell. The lower panel represents the best least-square fit of the integrated heat obtained after subtracting the heat of dilution. (TIF) [file ppat.1008489.s004.tif]

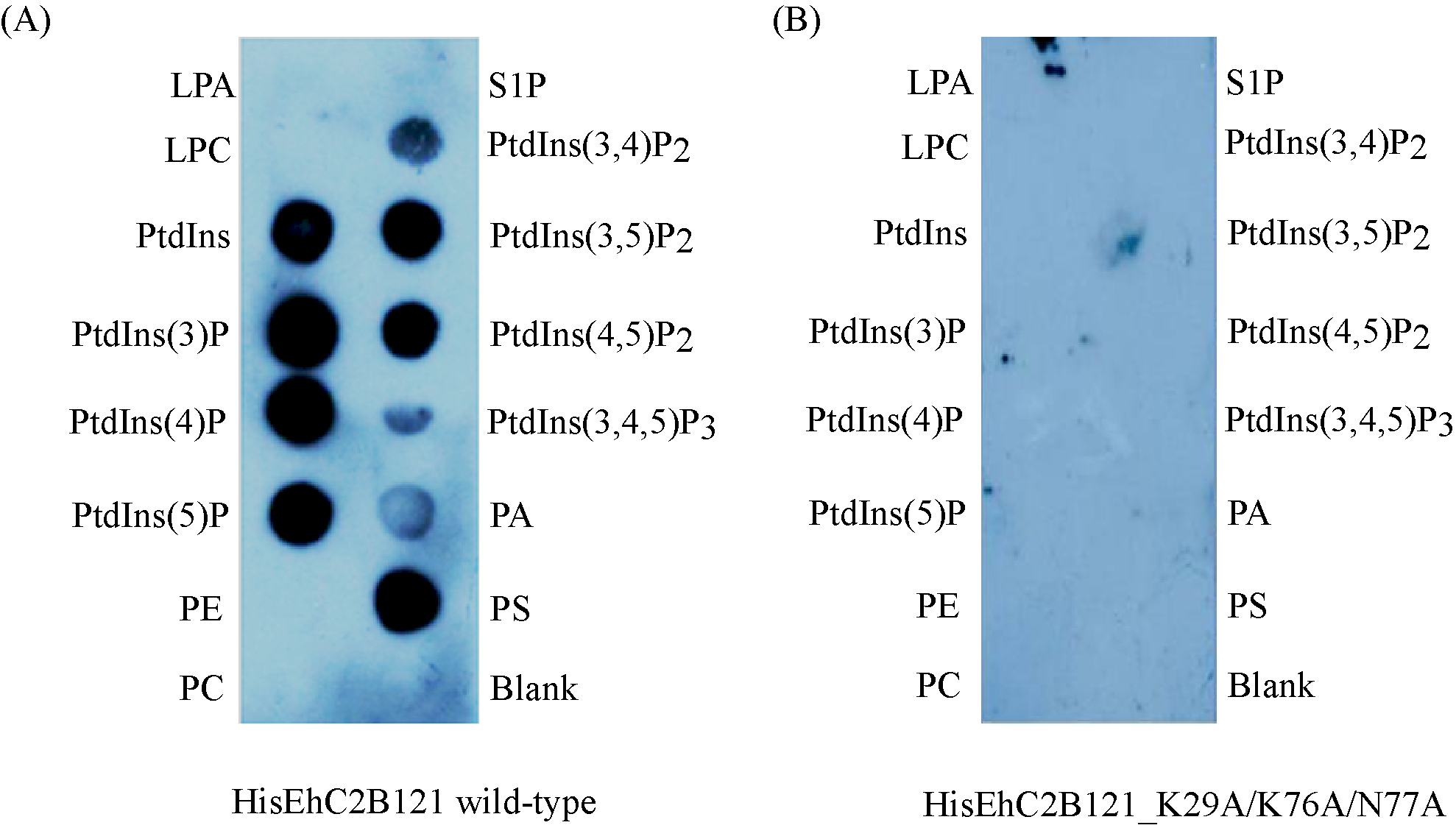

Supplement: S5 Fig — Nitrocellulose membrane with different lipids spots was blocked by 5% BSA prepared in TBST and then incubated with (A) wild-type and (B) lipid-mutants HisEhC2B121 for 4 hours at room temperature. The binding of the C2 domain protein with lipids was detected by immunoblotting by using an anti-His antibody. (TIF) [file ppat.1008489.s005.tif]

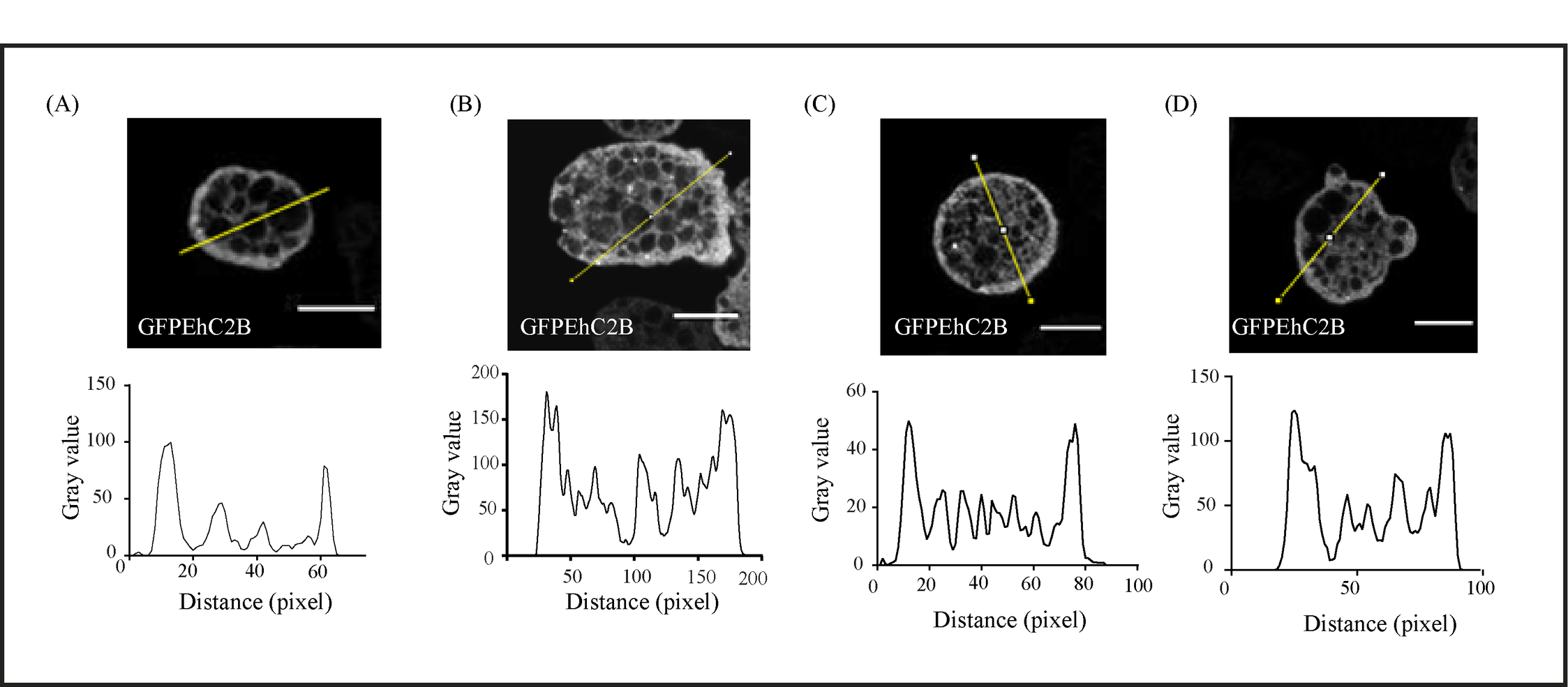

Supplement: S6 Fig — The fluorescence intensity of GFPEhC2B was analysed by drawing a line across multiple amoebic trophozoites and change in the fluorescence intensity with the distance is shown by line intensity plot. (TIF) [file ppat.1008489.s006.tif]

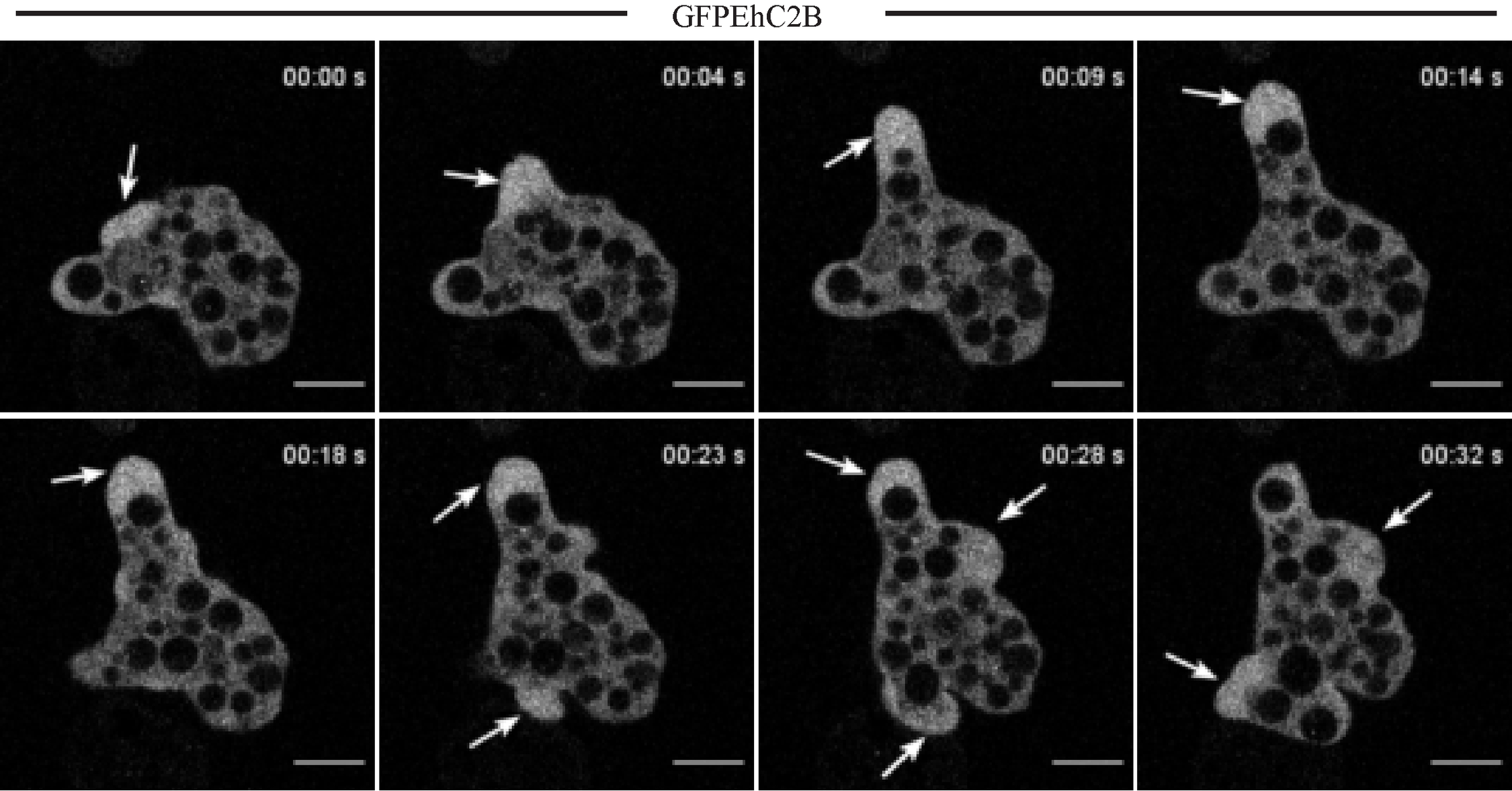

Supplement: S7 Fig — The montage shows the abundance of GFPEhC2B beneath the membrane as indicated by white colour arrows. (TIF) [file ppat.1008489.s007.tif]

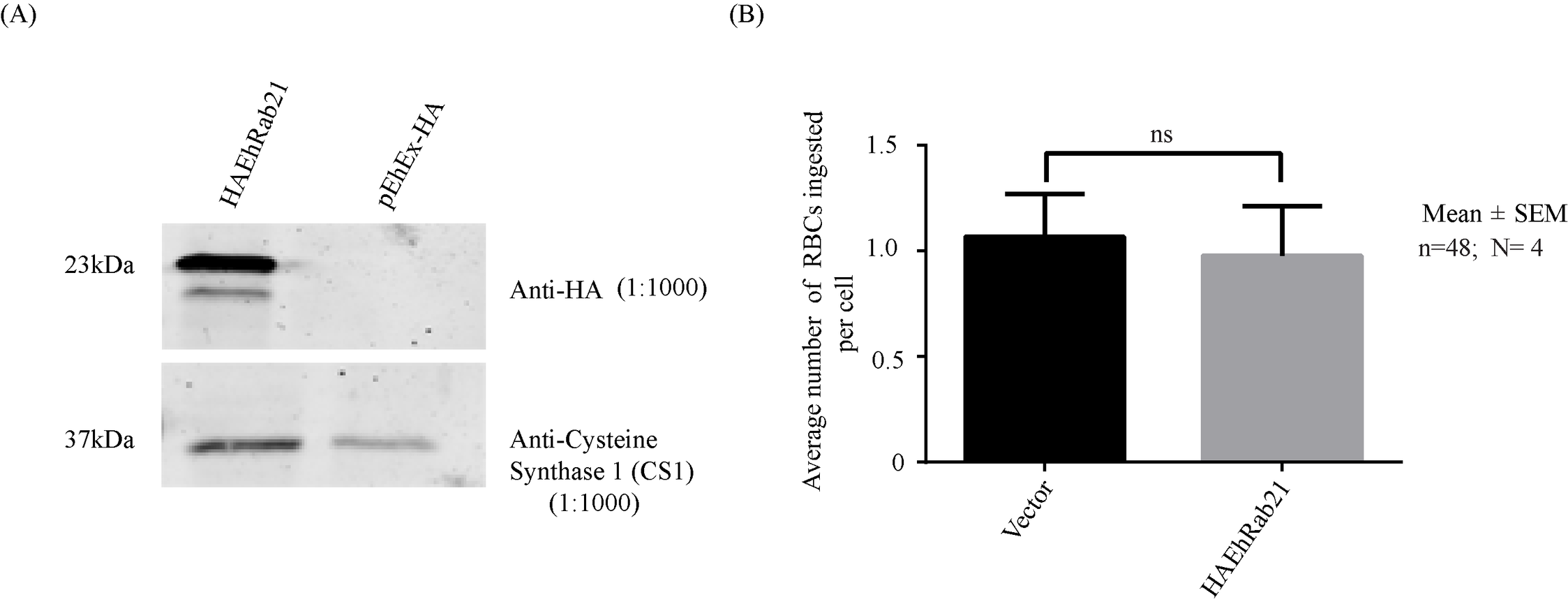

Supplement: S8 Fig — (A) Total cell lysates (100μg) from pEhEx-HA (Vector control) and pEhEX-HAEhRab21 transfected amoebic trophozoites were analysed on SDS-PAGE followed by immunoblotting using anti-HA (1:1000) antibody. For loading control anti-EhCS1 (1:1000) antibody was used. The blot was then probed with secondary antibodies, anti-mouse (1:10000) and anti-rabbit (1:1000) respectively. (B) Amoebic trophozoites were stably expressing pEhEx-HAEhRab21were incubated with Cell Tracker Orange-labelled RBCs (1:50) for 10 mins at 37°C. Trophozoites were then fixed, permeabilised, stained and later studied by confocal microscopy. The RBCs ingested per cell were enumerated manually for n = 48 cells from N = 4 set of biological repeats. The data represents Mean±SEM; ns-non significant. (TIF) [file ppat.1008489.s008.tif]

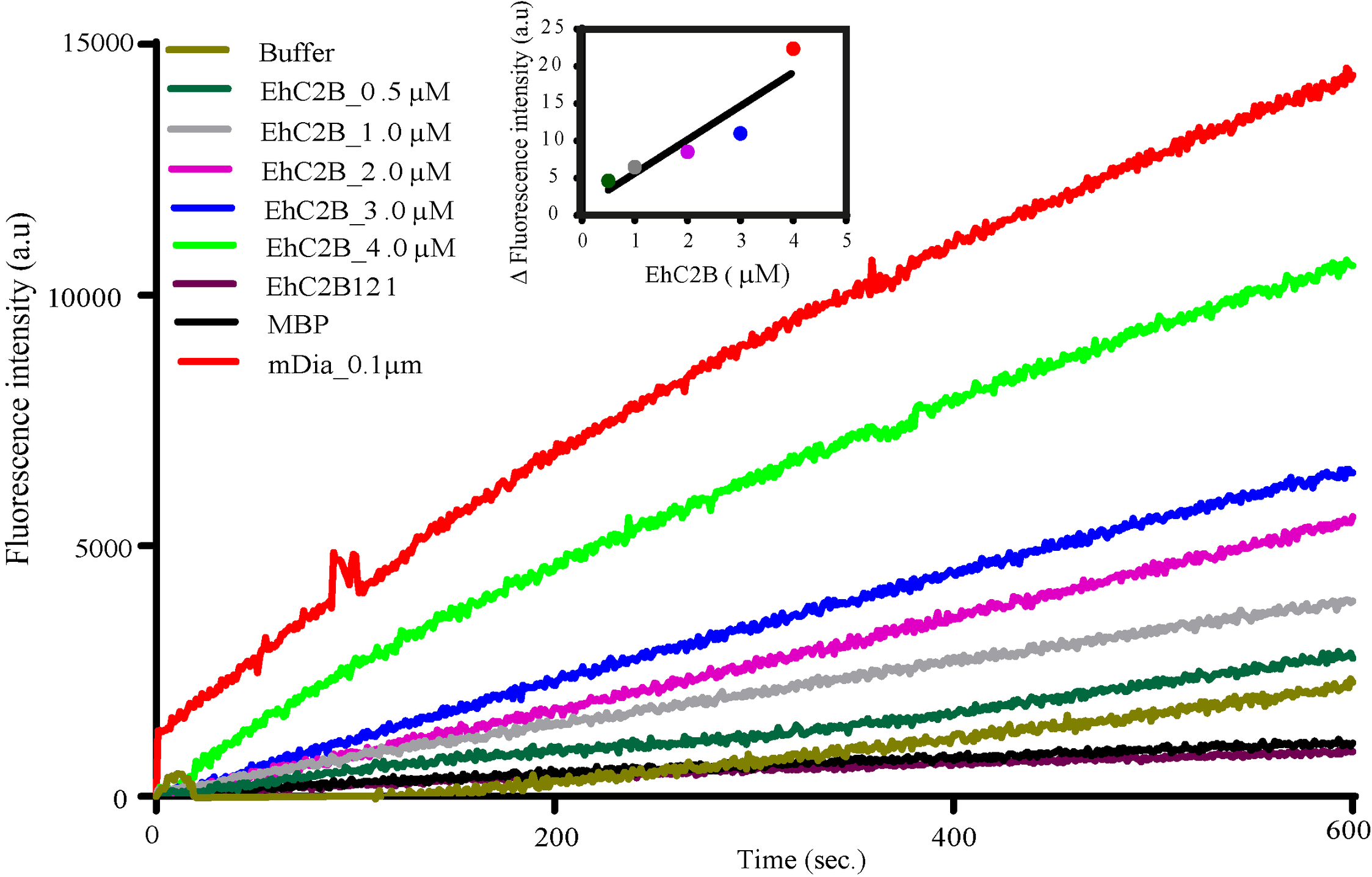

Supplement: S9 Fig — Pyrene labelled monomeric rabbit muscles actin (0.250 μM) was allowed to polymerize in the presence of different concentrations of EhC2B or EhC2B121, mDia1, and MBP as mentioned in detail in “Materials and methods” section. The fluorescence intensity of polymerization was measured at 360 and 407nm of excitation or emission spectra, respectively. (TIF) [file ppat.1008489.s009.tif]
